# Associations between cervical intraepithelial neoplasia during pregnancy, previous excisional treatment, cone-length and preterm delivery: a register-based study from western Sweden

**DOI:** 10.1186/s12916-022-02276-6

**Published:** 2022-02-22

**Authors:** Johanna Wiik, Cecilia Kärrberg, Staffan Nilsson, Björn Strander, Bo Jacobsson, Verena Sengpiel

**Affiliations:** 1 Department of Obstetrics and Gynecology, Institute of Clinical Sciences, Sahlgrenska Academy, University of Gothenburg, Gothenburg, Sweden; 2 Department of Gynecology and Obstetrics, Østfold Hospital Trust, Kalnes, Norway; 3 Region Västra Götaland, Department of Obstetrics and Gynecology, Sahlgrenska University Hospital, Gothenburg, Sweden; 4 Regional Cancer Centre West, Region Västra Götaland, Gothenburg, Sweden; 5 Department of Laboratory Medicine, Institute of Biomedicine, Sahlgrenska Academy, University of Gothenburg, Gothenburg, Sweden; 6 Department of Mathematical Sciences, Chalmers University of Technology, Gothenburg, Sweden; 7 Department of Genetics and Bioinformatics, Division of Health Data and Digitalisation, Institute of Public Health, Oslo, Norway

**Keywords:** Preterm delivery, Preterm prelabor rupture of the membranes, Cone-length, Cervical intraepithelial neoplasia

## Abstract

**Background:**

Excisional treatment of cervical intraepithelial neoplasia (CIN) has been associated with increased risk of preterm delivery (PTD), although the underlying mechanism is as yet unclear. Studies on formalin-fixed excised tissue indicate that the risk increases with cone-length, but the magnitude of increase is uncertain, especially in case of minor excisions (≤10 mm), as well compared to women with untreated CIN during pregnancy. This study assesses the impact of cone-length at previous treatment for CIN as well as diagnosis of CIN during pregnancy on the risk of PTD.

**Methods:**

A register-based cohort study in western Sweden linking cervical cytology, histology, and treatment data from the Swedish National Cervical Screening Registry to data on obstetric outcomes in singleton pregnancies 2008–2016 from the Swedish Medical Birth Registry. These groups were compared for PTD and other obstetric outcomes: (1) women with one excisional treatment (*n*=3250, including a subgroup (*n*=2408) with cone-length measured before fixation; (2) women with untreated CIN diagnosed during pregnancy (*n*=1380); and (3) women with normal cytology (*n*=42,398). Logistic regression analyses were adjusted for socioeconomic and health-related confounders.

**Results:**

Treated women had increased risk of PTD (adjusted odds ratio (aOR) 1.60, 95% confidence interval (CI) 1.21–2.12), spontaneous PTD (aOR 1.95, 95% CI 1.40–2.72) and preterm prelabor rupture of membranes (pPROM) (aOR 2.74, 95% CI 1.66–4.51) compared to the CIN during pregnancy group. ORs were similar when compared to the normal cytology group. Risks of these outcomes increased with cone-length. Mean cone-length was 9.1 mm. Cone-length ≤10 mm was associated with increased risk of PTD (aOR 1.41, 95% CI 1.02–1.94), spontaneous PTD (aOR 1.73, 95% CI 1.18–2.54), and pPROM (aOR 2.44, 95% CI 1.40–4.28), compared to the CIN during pregnancy group. The PTD risk was similar for cone-lengths 3–10 mm, thereafter increasing by 15% with each additional millimeter.

**Conclusions:**

This study suggests that all excisional treatment, including small cones, are associated with increased risk of PTD and pPROM. Risks increase further with cone-length. In women of reproductive age, clinicians should aim to remove all CIN but minimal healthy cervical tissue.

Cone-length should be recorded at treatment, for future prenatal risk estimation.

**Supplementary Information:**

The online version contains supplementary material available at 10.1186/s12916-022-02276-6.

## Background

Excisional treatment for cervical intraepithelial neoplasia (CIN) has been associated with preterm delivery (PTD) and preterm prelabor rupture of membranes (pPROM) in subsequent pregnancies [1–7]. Moreover, treatment has been associated with neonatal mortality and morbidity [3, 8]. PTD is the leading cause of neonatal mortality and short- and long-term morbidity [9, 10]. Studies have yielded contradicting results regarding the impact of excisional treatment on the risk of subsequent PTD. The choice of comparison group seems to affect the effect magnitude [7]. It has been suggested that women with untreated CIN also have increased risk of PTD [11–13] and that excisional treatment increases the risk further [3].

In a recent national register-based study in Sweden (1999–2016), we observed an increased risk of PTD, pPROM, and neonatal mortality in women with human papillomavirus (HPV) infection in conjunction with pregnancy. However, there was a stronger association between previous excisional treatment and these adverse obstetric outcomes [8]. We also observed an increased risk of infectious complications in subsequent pregnancies after excisional treatment, suggesting that ascending infection through the cervix might be involved in a pathway leading to PTD in these cases. However, the mechanism underlying the increased PTD risk after excisional treatment remains unclear. Hypotheses include immunomodulation related to HPV infection affecting parturition pathways and acquired mechanical weakness secondary to loss of cervical tissue [14–16]. The risk seems to be increased with techniques that remove or destroy larger portions of the cervix [3, 17, 18]. Some previous studies have found an increased risk of PTD with increasing cone-length at excision [3, 19–23] and a recent systematic review concluded that PTD risk increases with cone-length of the excised specimen [3]. A larger excised volume at treatment has also been associated with shorter cervix in asymptomatic pregnancies, measured with transvaginal ultrasound [24]. Second-trimester transvaginal ultrasound measurement of cervical length has been shown to predict spontaneous PTD with intermediate accuracy. It has been suggested that by adding the maternal obstetric history to the information about cervical length an improvement of the predictive accuracy might be achieved [25, 26].

With growing evidence suggesting that PTD risk is higher related to more radical techniques, there has been a tendency toward less aggressive treatment [4]. However, there is concern that smaller excisions could lead to a diminished decrease in the increased risk of future invasive cancer [27, 28]. It has not been established whether minor excisions (≤10 mm) increase the risk of PTD [3, 18], but some studies show no increased risk [20–22]. To the best of our knowledge, no study has yet examined the effect of different cone-lengths, compared to women with untreated CIN during pregnancy. This comparison enables a more unbiased estimate of the risk related to treatment, distinguishing it from the effects of HPV/CIN. Moreover, associations between cone-length and other adverse obstetric outcomes have not been studied in detail. One small study from New Zealand found an increased risk of pPROM associated with cone-length [21]. The issue of whether infectious complications in subsequent pregnancies are increased related to cone-length has not been studied so far.

Information about cone-length in earlier retrospective studies is based on histopathology reports on formalin-fixed samples, entailing a risk of underestimation due to specimen shrinkage. Ideally, cone-length should thus be assessed in fresh tissue. A unique opportunity to study these associations in a population-based manner exists in Sweden. Since 2008, cone-length is measured in a standardized way by the colposcopist before fixation, and recorded in the Process Register of the Swedish National Cervical Screening Registry (NKCx/Process) [29].

In summary, excisional treatment for CIN increases the risk of PTD in subsequent pregnancies, but the underlying mechanism is still not understood. Several studies found increasing PTD risk with cone-lengths. It is unclear whether the PTD risk also increases after minor excisions (≤10 mm) or in women with untreated CIN during pregnancy.

This study aimed at exploring the impact of excisional treatment for CIN on the risk of PTD and other adverse obstetric and neonatal outcomes, compared to women with normal cervical cytology history and to women with CIN during pregnancy, as well as at investigating how these risks were related to cone-length at treatment.

## Methods

This is a population-based study utilizing data from several Swedish health and quality registers. Data were linked based on the unique personal identification number held by each resident of Sweden.

### Data sources

1. The Swedish Medical Birth Register (MBR), established in 1973 and maintained by the Swedish National Board of Health and Welfare, a mandatory register comprising all births in Sweden from 22 completed weeks of gestation and up [30].
2. The Swedish National Cervical Screening Registry/Analysis (NKCx/Analysis), a quality register established in 1978, with full national coverage of all cervical cytology results since 1997 and full national coverage of histology results since 1998 [31].
3. NKCx/Process is the process register within the NKCx, first established in the western region of Sweden. It contains real-time data on screening invitations, as well as histology, cytology, and HPV results. It also includes data, from the western health care region of Sweden, on cervical dysplasia treatments beginning in 2002 and on cone-length beginning in 2008 [29]. Cone-lengths are measured, before fixation, in a standardized manner by colposcopists (Additional file 1: Fig. S1) [32].
4. The Swedish Cancer Register, a mandatory register established in 1958 and maintained by the Swedish National Board of Health and Welfare, registering all cancer and CIN3 diagnoses in Sweden [33].
5. Data on the country of birth, education level, and income were obtained from the Swedish Register of Education [34], the Total Population Register [35], and the Income and Tax Assessment Register [36], maintained by Statistics Sweden (SCB).

This study was reported according to the Strengthening the Reporting of Observational Studies in Epidemiology guidelines (Additional file 2: STROBE Checklist). The study was prospectively planned in 2015, the dataset was retrieved in 2019, and analyses were conducted until July 2021. The analysis plan has not been published but overall exposures, outcomes, confounders, and analyses were planned in 2015 by the research team, based on hypotheses drawn from previous studies. After obtaining and reviewing the database contents, but before starting the analyses, the final definition of study groups and outcomes was determined based on the available data and the quality of the dataset.

### Study population

All women with singleton births between January 1, 2008, and December 31, 2016, registered in the MBR were identified. Women with a history of chronic inflammatory disease, organ transplantation, or human immunodeficiency virus infection were excluded (see Additional file 3: Table S1 for the International Statistical Classification of Diseases and Related Health Problems -10th revision (ICD-10) codes leading to exclusion).

**Fig. 1:**
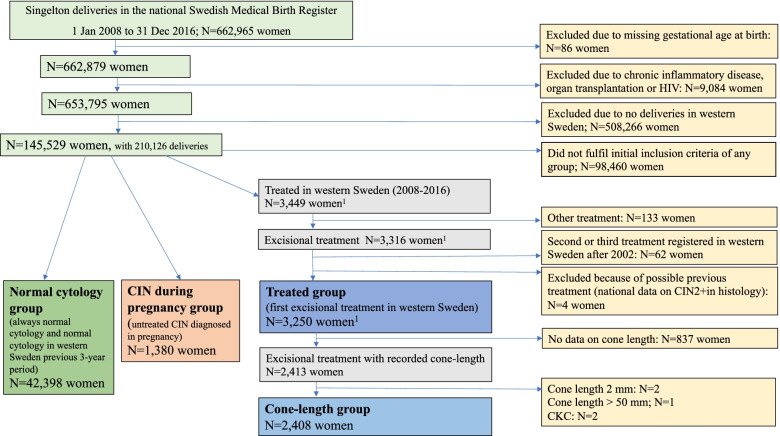
Flowchart of the study population. The different study groups were the normal cytology group, the CIN during pregnancy group, and the treated group with the subgroup cone-length group. Inclusion into the groups was based on the history of cervical cytology, cervical histology, previous treatment for CIN, and recorded cone-length at treatment. ^1^ *n*=158 women had a previous delivery in the CIN during the pregnancy group and were excluded from the treated group when compared to the CIN during the pregnancy group. CIN, cervical intraepithelial neoplasia; CKC; cold-knife conization; HIV, human immunodeficiency virus; *N*, number

Forty-six thousand eight hundred seventy women had at least one delivery fulfilling the exposure criteria for inclusion in one of the study groups (Fig. 1). Together with women that did not fulfill the exposure criteria for study group inclusion, and that did have a singleton delivery recorded in western Sweden in the MBR during this time period, they comprised a population of 145,529 women with 210,126 singleton deliveries.

### Exposures

A woman with a singleton delivery registered in the MBR 2008–2016 was eligible for inclusion in one of the study groups if the exposure criteria were fulfilled, according to NKCx and Swedish Cancer Register data. Women with treatment recorded in NKCx/Process before 2008 were excluded from all study groups. Furthermore, women with histological diagnoses of CIN2 or of more severe lesions (CIN2+) before 2008 in the NKCx/Analysis and the Swedish Cancer Register were excluded, since they might have undergone excisional treatment (Fig. 1). For an exact description of study groups, see Additional file 3: Table S2 [37].

The study groups were defined as follows:

1. *Treated group*: cervical excisional treatment performed in western Sweden between Jan 1, 2008, and Dec 31, 2016, and registered in NKCx/Process, and no previous excisional treatment (*n* = 3250). The first delivery after treatment was included in the analyses.
  1b) *Cone-length group*: subgroup of the treated group, treated with loop electrosurgical excision procedure (LEEP) (also known as large loop excision of the transformation zone (LLETZ))) or with laser conization, and with recorded cone-length (*n* = 2408). Cone-length <3 mm or >50 mm at treatment were excluded due to suspected misreporting. Two women who had undergone cold knife conization (CKC) were excluded to get more homogenous treatment data and facilitate interpretation.
2. *CIN during pregnancy group*: histologically diagnosed CIN or adenocarcinoma in situ (AIS) at cervical biopsy during pregnancy in western Sweden, registered in NKCx/Process, and with no treatment for CIN before the included delivery (*n*=1380). The first eligible delivery was included.
3. *Normal cytology group*: lifetime history of exclusively normal cervical cytology results in NKCx/Analysis, and at least one sample taken in western Sweden and registered in NKCx/Process during the three years preceding the included delivery (*n*=42,398). Only one delivery per woman, chosen at random, was included in the analyses.

There were 158 women with a delivery included in the CIN during the pregnancy group who also had a subsequent delivery included in the treated group. One hundred fifteen of these had recorded cone-lengths. These women were excluded from the treated group when it was compared to the CIN during the pregnancy group.

### Outcomes

The primary outcome was PTD at 22+0–36+6 weeks (154–258 days) of gestation, with subanalyses for early PTD (22–33 weeks (154–237 days) of gestation) and very early PTD (22–27 weeks (154–195 days) of gestation). Gestational age was retrieved from the MBR based on the best estimate, i.e., ultrasound determination when available and last menstrual period or estimation of gestational age at the delivery ward in the remaining cases.

Secondary outcomes were pPROM (determined according to ICD-10 codes in the MBR) and spontaneous PTD (a delivery starting with pPROM or preterm labor, excluding preterm deliveries that started with induction or cesarean section).

Additional outcomes comprised prelabor rupture of membranes (PROM) in term pregnancies (≥37 weeks of gestation), chorioamnionitis, and neonatal sepsis. Furthermore, comparisons were also made between the study groups concerning intrauterine fetal death, neonatal mortality (1–28 days), Apgar score <7 at 5 min, small for gestational age (SGA) (birthweight less than − 2 standard deviations (SD) according to Swedish reference curves) [38] and intrapartum fever (see Additional file 3: Table S3 for outcome definitions).

### Background variables

Multivariate analyses were adjusted for the following variables retrieved from the prenatal care records in the MBR and SCB registers: year of delivery (2008–2010, 2011–2013, 2014–2016), maternal age at delivery (<23, 23–30, 31–38, >38), body mass index (BMI) (underweight (<18.5), normal-weight (18.5–24.9), overweight (25–29.9), obese (≥30), missing), parity (0, 1–3, >3), marital status (cohabiting, single, other, missing), country of birth (Sweden, Europe, Asia, America/Oceania, Africa, other/unknown), infant’s sex (boy/girl), smoking (never, before pregnancy, in early pregnancy only, in the third trimester, missing), highest disposable household income during the 3 years preceding delivery (population divided into tertiles for every year), education level at delivery (primary, secondary, post-secondary <3 years, post-secondary ≥3 years, missing), and assisted reproduction (yes/no). Adjustments were based on a priori knowledge of risk factors for PTD [39–41]. Furthermore, data on employment at time of delivery, chronic renal disease, diabetes, epilepsy, and chronic hypertension were also collected.

Data registered in western Sweden concerning histological diagnosis and type of treatment were retrieved from NKCx/Process (Additional file 3: Table S4). If several diagnoses had been recorded, the most serious was included.

### Statistical analyses

Analyses were performed with the “R” data analysis tool (version 4.0.0, The R Foundation for Statistical Computing, Vienna, Austria, https://www.r-project.org/) and SPSS software (version 26.0, IBM, https://www.ibm.com/analytics/spss-statistics-software). A significance level of 0.05 was applied throughout. Descriptive data are presented with numbers and percentages for categorical variables and with mean and standard deviation (SD) and median and interquartile range (IQR) for continuous variables.

The normal cytology group, the treated group, and the CIN during pregnancy group were compared, regarding obstetric and neonatal outcomes, by unadjusted and adjusted logistic regression analysis.

In a subgroup analysis, the treated group with benign histology was compared to the normal cytology group regarding risk of spontaneous PTD and pPROM. Furthermore, stratified analyses were used to compare PTD risk, within the CIN during pregnancy group, in women with high-grade lesions to women with low-grade lesions.

#### Cone-length and obstetric and neonatal outcomes

Unadjusted and adjusted logistic regression analyses were performed to compare the normal cytology group to the cone-length subgroups categorized as follows: 3–5 mm, 6–9 mm, 10 mm, ≤10 mm, 11–12 mm, 13–15 mm, and >15 mm. Outcomes in the subgroup with cone- lengths ≤10 mm were also compared to outcomes in the CIN during the pregnancy group with unadjusted and adjusted logistic regression analyses, including a subgroup analysis exclusively of women with histologically diagnosed high-grade lesions.

Associations between cone-length and PTD, spontaneous PTD, pPROM, PROM, chorioamnionitis, and neonatal sepsis were studied with adjusted and unadjusted binary logistic regression in all women with recorded cone-lengths, only in women with cone-lengths ≤10 mm and in all women after cone-lengths <10 mm had been truncated to 10 mm. Moreover, using the truncated cone-lengths, three logistic regression models were fit for the outcomes PTD, spontaneous PTD, and pPROM in a standardized woman (age 23–30, nullipara, non-smoker, delivery of a male child during the period 2014–2016), with the left truncated cone-length as predictor, and presented graphically.

## Results

### Characteristics of the study population

Women in the CIN during the pregnancy group were younger than those in the other groups. There were fewer nulliparas in the normal cytology group (48%) than in the CIN during the pregnancy group (58%) and the treated group (60%). The treated group had given birth more frequently in 2014-2016 than the other groups. Women in the normal cytology group were more frequently non-smokers. There were fewer pregnancies after assisted reproduction in the CIN during the pregnancy group than in the other groups (Table 1).

**Table 1:** Demographics and clinical characteristics in the study groups

Characteristics	Normal cytology group (*N*=42,398)	CIN during pregnancy group (*N*=1380)	Treated group (*N*=3250)
	*n* (%)	*n* (%)	*n* (%)
*Background factors adjusted for in the adjusted analyses*			
**Age at delivery (years)**			
< 23	247 (0.6)	17 (1.2)	13 (0.4)
23–30	24,485 (57.8)	862 (62.5)	1720 (52.9)
31–38	16,438 (38.8)	460 (33.3)	1393 (42.9)
>38	1228 (2.9)	41 (3.0)	124 (3.8)
**BMI class (kg/m**^**2**^**)**			
Underweight (<18.5)	874 (2.1)	24 (1.7)	73 (2.2)
Normal-weight (18.5–24.9)	23,957 (56.5)	815 (59.1)	1915 (58.9)
Overweight (25–29.9)	9144 (21.6)	256 (18.6)	654 (20.1)
Obese (≥30)	4110 (9.7)	113 (8.2)	287 (8.8)
Missing	4313 (10.2)	172 (12.5)	321 (9.9)
**Smoking**			
Never	34,969 (82.5)	947 (68.6)	2464 (75.8)
Before pregnancy	3063 (7.2)	197 (14.3)	346 (10.6)
In early pregnancy only	727 (1.7)	40 (2.9)	82 (2.5)
In the third trimester	1123 (2.6)	107 (7.8)	170 (5.2)
Missing	2516 (5.9)	89 (6.4)	188 (5.8)
**Infant’s sex**			
Boy	21,721 (51.2)	728 (52.8)	1646 (50.6)
Girl	20,676 (48.8)	652 (47.2)	1604 (49.4)
**Pregnant by assisted reproduction**			
Yes	1276 (3.0)	15 (1.1)	122 (3.8)
**Parity**			
0	20,765 (49.0)	801 (58.0)	1964 (60.4)
1–3	21,366 (50.4)	568 (41.2)	1269 (39.0)
>3	267 (0.6)	11 (0.8)	17 (0.5)
**Marital status**			
Cohabiting	37,642 (88.8)	1175 (85.1)	2853 (87.8)
Single	494 (1.2)	33 (2.4)	53 (1.6)
Other	1149 (2.7)	64 (4.6)	97 (3.0)
Missing	3113 (7.3)	108 (7.8)	247 (7.6)
**Education level**^**a**^			
Primary school, up to 9 years	2785 (6.6)	128 (9.3)	218 (6.7)
Secondary	14,421 (34.0)	618 (44.8)	1246 (38.3)
Post-secondary, <3 years	5765 (13.6)	207 (15.0)	527 (16.2)
Post-secondary, ≥3 years	19,166 (45.2)	413 (29.9)	1250 (38.5)
Missing	261 (0.6)	14 (1.0)	9 (0.3)
**Country of birth**			
Sweden	35,031 (82.6)	1183 (85.7)	2948 (90.7)
Europe	3015 (7.1)	102 (7.4)	154 (4.7)
Asia	2908 (6.9)	64 (4.6)	96 (3.0)
America/Oceania	480 (1.1)	14 (1.0)	37 (1.1)
Africa	954 (2.3)	17 (1.2)	14 (0.4)
Unknown/missing	10 (0.0)	0	1 (0.0)
**Year of delivery**			
2008–2010	12,590 (29.7)	308 (22.3)	349 (10.7)
2011–2013	12,831 (30.3)	440 (31.9)	1128 (34.7)
2014–2016	16,977 (40.0)	632 (45.8)	1773 (54.6)
**Highest disposable household income, 3 years preceding delivery**			
Lowest tertile	6963 (16.4)	267 (19.3)	465 (14.3)
Middle tertile	13,503 (31.8)	485 (35.1)	1060 (32.6)
Highest tertile	21,932 (51.7)	628 (45.5)	1725 (53.1)
*Other background factors*			
**Employment**			
Full-time	22,135 (52.2)	727 (52.7)	1937 (59.6)
Part-time	9238 (21.8)	273 (19.8)	587 (18.1)
None	7003 (16.5)	238 (17.2)	387 (11.9)
Missing	4022 (9.5)	142 (10.3)	339 (10.4)
**Intercurrent disease**^**b**^			
Renal disease	140 (0.3)	7 (0.5)	12 (0.4)
Diabetes	204 (0.5)	6 (0.4)	11 (0.3)
Epilepsy	109 (0.3)	4 (0.3)	11 (0.3)
Chronic hypertension	84 (0.2)	4 (0.3)	8 (0.2)
**Gestational age estimated by**			
Ultrasound	38,911 (91.8)	1266 (91.7)	2997 (92.2)
Last menstrual period	505 (1.2)	12 (0.9)	31 (1.0)
Other^c^	2982 (7.0)	102 (7.4)	222 (6.8)

Data are presented as numbers (percentages)

Percentages are based on those with available data. Percentages of missing are based on the total numbers

^a^Highest education level at time of delivery

^b^As reported in prenatal care records. Missing values were interpreted as lacking any intercurrent disease, in accordance with how data are registered in records

^c^Ultrasound, last menstrual period, and/or estimation of gestational age at the delivery ward

*BMI* body mass index, *CIN* cervical intraepithelial neoplasia, *SD* standard deviation

In the cone-length group, the majority (*n*=2055 (85%)) were treated with LEEP. Cone-lengths varied between 3 and 31 mm (mean 9.02, SD 2.99) The women treated with laser had longer cones (mean 9.53 mm, SD 3.36) than those treated with LEEP (mean 9.02, SD 2.92) (*p*=0.003).

In the cone-length group, histology in the excised specimen was classified as follows; cancer (1.0%), high-grade lesions (74.5%), low-grade lesions (12.5%), dysplasia not further specified, signs of HPV infection (2.5%), benign (7.8%), and not classified/missing (1.7%).

### Associations between CIN during pregnancy, previous excisional treatment, and obstetric and neonatal outcomes

In the total dataset of 210,126 singleton deliveries in 145,529 women in western Sweden in 2008–2016, 9920 (4.7%) were PTD, 6699 (3.2 %) were spontaneous PTD, 2782 (1.3%) were pPROM, and 10,389 were deliveries after PROM at term (4.9% of all deliveries and 5.2% of term pregnancies). Of these deliveries, 537 (0.3%) were diagnosed with chorioamnionitis and 2525 (1.2%) with neonatal sepsis.

Mean (SD) and median (IQR) gestational age were 279.5 (12.4) and 281 (274–287) in the normal cytology group, 279.1 (13.3) and 281 (274–287) in the CIN during pregnancy group, and 277.3 (15.2) and 280 (272–286) in the treated group. The CIN during pregnancy group had an increased risk of PTD, compared to the normal cytology group; this increase was, however, no longer significant in the adjusted analyses (Table 2). When women with low-grade lesions were compared to women with high-grade lesions in the CIN during the pregnancy group, there was no significant difference in risk of PTD, (aOR 0.90, 95% CI 0.55–1.47, *p*=0.67).

**Table 2:** Adverse obstetric and neonatal outcomes in the CIN during pregnancy group and the treated group, compared to the normal cytology group, unadjusted and adjusted multivariable logistic regression analyses

Outcome	Normal cytology group (*N*=42,398)	CIN during pregnancy group (*N*=1380)	Unadjusted analyses		Adjusted analyses^a^		Treated group (*N*=3250)	Unadjusted analyses		Adjusted analyses^a^	
	*n* (%)	*n* (%)	OR (95% CI)	*p* value	aOR (95% CI)	*p* value		OR (95% CI)	*P* value	aOR (95%CI)	*p* value
PTD, <37 weeks	1794 (4.2)	74 (5.4)	1.28 (1.01–1.63)	**0.041**	1.21 (0.95–1.55)	0.12	244 (7.5)	1.84 (1.60–2.11)	**<0.001**	1.75 (1.51–2.01)	**<0.001**
Early PTD, <34 weeks	488 (1.2)	15 (1.1)	0.94 (0.56–1.58)	0.83	0.88 (0.52–1.49)	0.64	75 (2.3)	2.03 (1.59–2.59)	**<0.001**	1.93 (1.50–2.49)	**<0.001**
Very early PTD, <28 weeks	91 (0.2)	6 (0.4)	2.03 (0.89–4.65)	0.09	1.99 (0.86–4.61)	0.11	15 (0.5)	2.16 (1.25–3.73)	**0.006**	2.06 (1.17–3.63)	**0.012**
Spontaneous PTD	1264 (3.0)	49 (3.6)	1.20 (0.90–1.60)	0.22	1.13 (0.84–1.51)	0.43	194 (6.0)	2.07 (1.77–2.41)	**<0.001**	2.00 (1.70–2.34)	**<0.001**
pPROM	479 (1.1)	20 (1.4)	1.29 (0.82–2.02)	0.27	1.22 (0.78–1.92)	0.39	105 (3.2)	2.92 (2.36–3.62)	**<0.001**	2.63 (2.11–3.28)	**<0.001**
PROM, delivery at ≥ 37 weeks	2084 (5.1)	69 (5.3)	1.03 (0.81–1.32)	0.81	0.95 (0.74–1.21)	0.66	208 (6.9)	1.37 (1.19–1.59)	**<0.001**	1.23 (1.06–1.43)	**0.008**
SGA^b^	894 (2.1)	35 (2.5)	1.21 (0.86–1.70)	0.28	1.11 (0.78–1.56)	0.57	65 (2.0)	0.95 (0.74–1.22)	0.68	0.89 (0.69–1.15)	0.37
Apgar score <7 at 5 min	476 (1.1)	16 (1.2)	1.03 (0.63–1.71)	0.90	0.99 (0.60–1.64)	0.97	37 (1.1)	1.01 (0.72–1.42)	0.94	0.98 (0.70–1.38)	0.90
Neonatal mortality	43 (0.1)	1 (0.1)	0.71 (0.10–5.19)	0.74	0.70 (0.10–5.17)	0.73	2 (0.1)	0.61 (0.15–2.51)	0.49	0.66 (0.16–2.75)	0.57
Intrauterine fetal death	88 (0.2)	2 (0.1)	0.70 (0.17–2.84)	0.62	0.66 (0.16–2.70)	0.56	7 (0.2)	1.04 (0.48–2.24)	0.93	1.07 (0.49–2.34)	0.87
Chorioamnionitis	97 (0.2)	5 (0.4)	1.59 (0.64–3.90)	0.32	1.43 (0.58–3.54)	0.44	14 (0.4)	1.89 (1.08–3.31)	**0.027**	1.57 (0.89–2.80)	0.12
Intrapartum fever	300 (0.7)	9 (0.7)	0.92 (0.47–1.79)	0.81	0.77 (0.39–1.50)	0.44	20 (0.6)	0.87 (0.55–1.37)	0.54	0.68 (0.43–1.07)	0.09
Neonatal sepsis	591 (1.4)	22 (1.6)	1.15 (0.75–1.76)	0.53	1.01 (0.66–1.56)	0.95	64 (2.0)	1.42 (1.10–1.84)	**0.008**	1.20 (0.92–1.56)	0.18

Statistically significant *p*-values in bold type

^a^Analyses adjusted for: year of delivery, maternal age, parity, BMI, marital status, country of birth, infant’s sex, smoking, income, education level and assisted reproduction

^b^Missing data: normal cytology group, *n*=27; CIN during pregnancy group, *n*=0; treated group, *n*=2

*aOR*, adjusted odds ratio; *CI*, confidence interval; *CIN*, cervical intraepithelial neoplasia; *N*, number; *OR* odds ratio; *pPROM*, preterm prelabor rupture of membranes; *PROM*, prelabor rupture of membranes; *PTD*, preterm delivery; *SGA*, small for gestational age

The treated group had an increased risk of PTD, spontaneous PTD, early PTD, pPROM, and PROM, compared to the normal cytology group (Table 2), as well as compared to the CIN during pregnancy group (Table 3). Compared to the normal cytology group, the treated group also had an increased risk of chorioamnionitis and neonatal sepsis; the increases were, however, no longer significant in the adjusted analyses (Table 2). Neonatal mortality and intrauterine fetal death were too rare to yield conclusive results.

**Table 3:** Adverse obstetric and neonatal outcomes in the treated group, compared to the CIN during pregnancy group, unadjusted and adjusted multivariable logistic regression analyses

Outcome	CIN during pregnancy group (*N*=1380)	Treated group (*N*=3092)^a^	Unadjusted analyses		Adjusted analyses^b^	
	*n* (%)	*n* (%)	OR (95% CI)	*p* value	aOR (95% CI)	*p* value
PTD <37 weeks	74 (5.4)	239 (7.7)	1.48 (1.13–1.93)	**0.004**	1.60 (1.21–2.12)	**0.001**
Early PTD <34 weeks	15 (1.1)	74 (2.4)	2.23 (1.28–3.90)	**0.005**	2.36 (1.32–4.23)	**0.004**
Very early PTD <28 weeks	6 (0.4)	15 (0.5)	1.12 (0.43–2.88)	0.82	0.90 (0.33–2.49)	0.84
Spontaneous PTD	49 (3.6)	189 (6.1)	1.77 (1.28–2.44)	**<0.001**	1.95 (1.40–2.72)	**<0.001**
pPROM	20 (1.4)	103 (3.3)	2.34 (1.45–3.80)	**0.001**	2.74 (1.66–4.51)	**<0.001**
PROM, delivery at ≥ 37 weeks	69 (5.3)	205 (7.2)	1.39 (1.05–1.84)	**0.022**	1.38 (1.03–1.85)	**0.030**
SGA^c^	35 (2.5)	63 (2.0)	0.80 (0.53–1.22)	0.30	0.88 (0.56–1.37)	0.57
Apgar score <7 at 5 min	16 (1.2)	37 (1.2)	1.03 (0.57–1.86)	0.92	1.00 (0.54–1.85)	1.00
Neonatal mortality	1 (0.1)	2 (0.1)	0.89 (0.08–9.85)	0.93	0.74 (0.06–9.07)	0.81
Intrauterine fetal death	2 (0.1)	7 (0.2)	1.56 (0.32–7.54)	0.58	1.62 (0.31–8.42)	0.57
Chorioamnionitis	5 (0.4)	14 (0.5)	1.25 (0.45–3.48)	0.67	1.35 (0.46–4.00)	0.58
Intrapartum fever	9 (0.7)	19 (0.6)	0.94 (0.43–2.09)	0.88	0.68 (0.29–1.57)	0.37
Neonatal sepsis	22 (1.6)	62 (2.0)	1.26 (0.77–2.06)	0.35	1.35 (0.81–2.25)	0.25

Statistically significant *p* values in bold type

^a^158 women also had a previous delivery in the CIN during pregnancy group and were excluded from the treated group in the analyses

^b^Analyses adjusted for: year of delivery, maternal age, parity, BMI, marital status, country of birth, infant’s sex, smoking, income, education level and assisted reproduction

^c^Missing data: CIN during pregnancy group, *n*=0, treated group, *n*=2

*aOR*, adjusted odds ratio; *CI*, confidence interval; *CIN*, cervical intraepithelial neoplasia; *N*, number; *OR*, odds ratio; *pPROM*, preterm prelabor rupture of membranes; *PROM*, prelabor rupture of membranes; *PTD*, preterm delivery; *SGA*, small for gestational age

When only women with benign histology in the treated group (*n*=271) were included, there was still an increased risk of spontaneous PTD (aOR 1.79, 95% CI 1.06 − 3.03, *p*=0.03), as well as of pPROM (aOR 3.04, 95% CI 1.60–5.79, *p*=0.001), compared to the normal cytology group. The corresponding increased risk of PTD (aOR 1.51, 95% CI 0.93–2.45, *p*=0.09) was, however, not significant. The risk of spontaneous PTD was similar in women treated with laser (*n*=24, 6.5%) and in women treated with LEEP (*n*=126, 6.1%) in the cone-length group.

### Associations between cone-length and adverse obstetric and neonatal outcomes

**Fig. 2:**
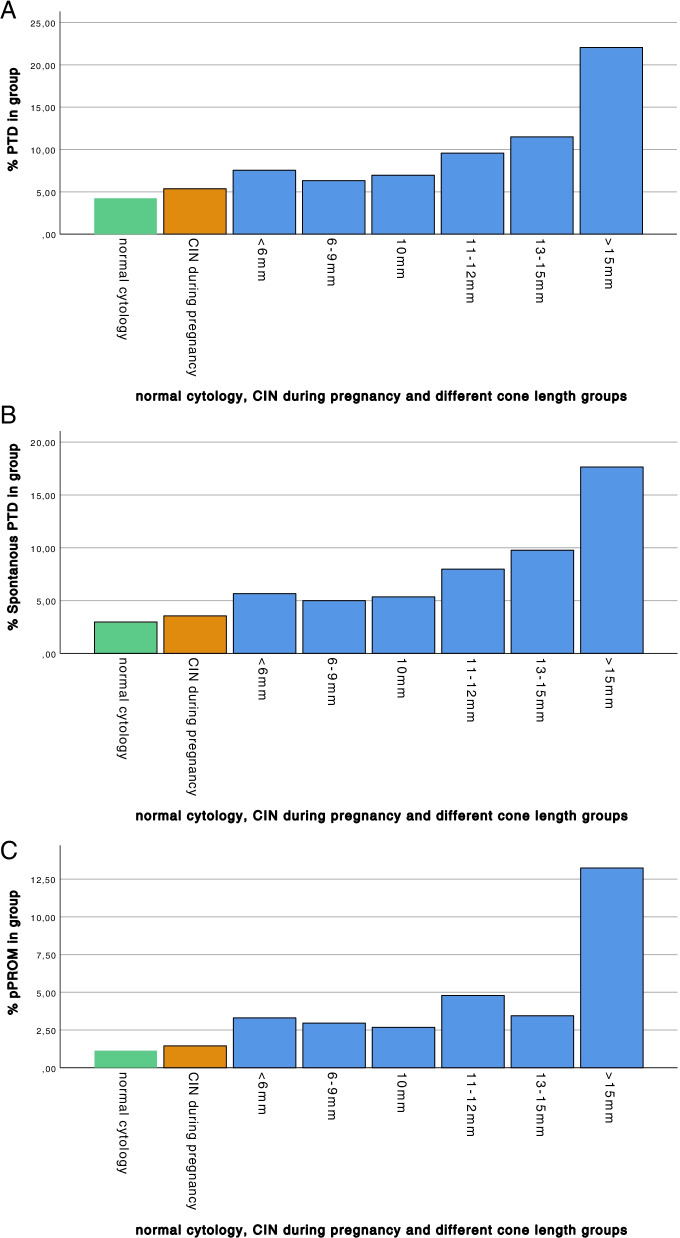
Incidence of PTD (**a**), spontaneous PTD (**b**), and pPROM (**c**) in the normal cytology, CIN during pregnancy and cone-length groups. **a** The percentage of PTD increased with cone-length. Number (percentage) of PTD in the normal cytology group, the CIN during pregnancy group and different cone length groups: normal cytology 1794/42,398 (4.2%), CIN during pregnancy 74/1380 (5.4%), <6 mm 16/212 (7.5%), 6–9 mm 77/1219 (6.3%), 10 mm 26/374 (7.0%), 11–12 mm 33/361 (9.1%), 13–15 mm 20/174 (11.5%), >15 mm 15/68 (22.1%). CIN, cervical intraepithelial neoplasia, PTD, preterm delivery. **b** The percentage of spontaneous PTD increased with cone-length. Number (percentage) of spontaneous PTD in the normal cytology group, the CIN during pregnancy group and different cone-length groups: normal cytology 1264 /42,398 (3.0%), CIN during pregnancy 49/1380 (3.6%), <6 mm 12/212 (5.7%), 6–9 mm 61/1219 (5.0%), 10 mm 20/374 (5.3%), 11–12 mm 27/361 (7.5%), 13–15 mm 17/174 (9.8%), >15 mm 12/68 (17.6%). CIN, cervical intraepithelial neoplasia, PTD, preterm delivery. **c** The percentage of pPROM was increased in women with large excisions. Number (percentage) of pPROM in the normal cytology group, the CIN during pregnancy group and different cone-length groups: normal cytology 479/42,398 (1.1%), CIN during pregnancy 20/1380 (1.4%), <6 mm 7/212 (3.3%), 6–9 mm 36/1219 (3.0%), 10 mm 10/374 (2.7%), 11–12 mm 15/361 (4.2%), 13–15 mm 6/174 (3.4%), >15 mm 9/68 (13.2%). CIN, cervical intraepithelial neoplasia; pPROM, preterm prelabor rupture of membranes

The percentage of PTD, spontaneous PTD, and pPROM increased with increasing cone-length (Fig. 2a–c). The number of cones >15 mm was low (*n*=68), but this group had a high rate of adverse obstetric outcomes.

The risk of PTD, spontaneous PTD, and pPROM increased with increasing cone-length, compared to women with normal cytology. Treated women with cone-lengths up to 10 mm also had an increased risk of PTD, spontaneous PTD, and pPROM, compared to the normal cytology group (Tables 4 and 5). For unadjusted analyses, see Additional file 3: Tables S5a-b.

**Table 4:** Adverse obstetric and neonatal outcomes in cone-length groups, compared to the normal cytology group, adjusted multivariable logistic regression analyses

	Normal cytology group *N*=42,398	Treated, cone-length ≤10 mm *N*=1805			Treated, cone-length 11–12 mm *N*=361			Treated, cone-length 13–15mm *N*=174			Treated, cone-length >15mm *N*=68		
	*N* (%)	*N* (%)	aOR^a^	*p*	*N* (%)	aOR^a^	*p*	*N* (%)	aOR^a^	*p*	*N* (%)	aOR^a^	*p*
PTD, <37 weeks	1794 (4.2)	119 (6.6)	1.53 (1.26–1.86)	**<0.001**	33 (9.1)	2.19 (1.52–3.15)	**<0.001**	20 (11.5)	2.79 (1.74–4.48)	**<0.001**	15 (22.1)	5.90 (3.28–10.59)	**<0.001**
Spontaneous PTD	1264 (3.0)	93 (5.2)	1.72 (1.38–2.15)	**<0.001**	27 (7.5)	2.58 (1.73–3.85)	**<0.001**	17 (9.8)	3.40 (2.05–5.65)	**<0.001**	12 (17.6)	6.63 (3.51–12.52)	**<0.001**
pPROM	479 (1.1)	53 (2.9)	2.33 (1.73–3.12)	**<0.001**	15 (4.2)	3.42 (2.01–5.82)	**<0.001**	6 (3.4)	2.80 (1.23–6.39)	**0.014**	9 (13.2)	11.68 (5.64–24.18)	**<0.001**
PROM, delivery at ≥ 37 weeks	2084 (5.1)	113 (6.7)	1.17 (0.96–1.43)	0.12	30 (9.1)	1.67 (1.14–2.45)	**0.009**	9 (5.8)	1.09 (0.55–2.15)	0.81	10 (18.9)	4.12 (2.04–8.29)	**<0.001**
Chorioamnionitis	97 (0.2)	7 (0.4)	1.29 (0.59–2.82)	0.52	1 (0.3)	1.01 (0.14–7.33)	0.99	0	–	–	1 (1.5)	6.06 (0.81–45.38)	0.080
Neonatal sepsis	591 (1.4)	31 (1.7)	1.02 (0.71–1.47)	0.92	5 (1.4)	0.87 (0.36–2.13)	0.77	3 (1.7)	1.12 (0.36–3.53)	0.85	6 (8.8)	5.72 (2.43–13.47)	**<0.001**

Statistically significant *p* values in bold type

^a^Analyses adjusted for year of delivery, maternal age, parity, BMI, marital status, country of birth, infant’s sex, smoking, income, education level, and assisted reproduction

*aOR*, adjusted odds ratio; *CI*, confidence interval; *mm*, millimeter; *N*, number; *OR*, odds ratio; *pPROM*, preterm prelabor rupture of membranes; *PROM*, prelabor rupture of membranes; *PTD*, preterm delivery

**Table 5:** Adverse obstetric and neonatal outcomes in cone-length groups up to 10 mm, compared to the normal cytology group, adjusted multivariable logistic regression analyses

	Normal cytology group *N*=42,398	Treated, cone-length <6 mm *N*=212			Treated, cone-length 6–9 mm *N*=1219			Treated, cone-length 10 mm *N*=374		
	*N* (%)	*N* (%)	aOR^a^	*p*	*N* (%)	aOR^a^	*p*	*N* (%)	aOR^a^	*p*
PTD, <37 weeks	1794 (4.2)	16 (7.5)	1.72 (1.03–2.89)	**0.038**	77 (6.3)	1.47 (1.16–1.87)	**0.002**	26 (7.0)	1.57 (1.05–2.36)	**0.028**
Spontaneous PTD	1264 (3.0)	12 (5.7)	1.83 (1.02–3.31)	**0.044**	61 (5.0)	1.68 (1.29–2.20)	**<0.001**	20 (5.3)	1.75 (1.11–2.76)	**0.017**
pPROM	479 (1.1)	7 (3.3)	2.55 (1.19–5.49)	**0.016**	36 (3.0)	2.36 (1.66–3.34)	**<0.001**	10 (2.7)	2.07 (1.09–3.92)	**0.026**
PROM, delivery at ≥ 37 weeks	2084 (5.1)	11 (5.6)	0.93 (0.50–1.71)	0.81	79 (6.9)	1.23 (0.97–1.55)	0.09	23 (6.6)	1.14 (0.74–1.75)	0.55
Chorioamnionitis	97 (0.2)	0	–	–	4 (0.3)	1.11 (0.40–3.05)	0.84	3 (0.8)	2.73 (0.85–8.76)	0.09
Neonatal sepsis	591 (1.4)	3 (1.4)	0.79 (0.25–2.48)	0.69	22 (1.8)	1.09 (0.71–1.68)	0.70	6 (1.6)	0.95 (0.42–2.14)	0.90

Statistically significant *p* values in bold type

^a^Analyses adjusted for: year of delivery, maternal age, parity, BMI, marital status, country of birth, infant’s sex, smoking, income, education level, and assisted reproduction

*aOR*, adjusted odds ratio; *CI*, confidence interval; *mm*, millimeter; *N*, number; *OR*, odds ratio; *pPROM*, preterm prelabor rupture of membranes; *PROM*, prelabor rupture of membranes; *PTD*, preterm delivery.

When treated women with cone-lengths up to 10 mm were compared to the CIN during the pregnancy group, we found an increased risk of PTD (aOR 1.41, 95% CI 1.02–1.94, *p*=0.038), spontaneous PTD (aOR 1.73, 95% CI 1.18–2.54, *p*=0.005), and pPROM (aOR 2.44, 95% CI 1.40–4.28, *p*=0.002), (Additional file 3: Table S6); this was also found in the subgroup analyses including only high-grade lesions (Additional file 3: Table S7).

The risk of PTD and spontaneous PTD was similar for cone-lengths up to 10 mm and increased with cone-length (Fig. 2a, b and Tables 4 and 5). Cone-length was associated with an increased risk of PTD, spontaneous PTD, pPROM, PROM, and neonatal sepsis in the logistic regression analyses (Additional file 3: Table S8). When only cone-lengths 3–10 mm were included in the analyses (*n*=1805) no risk increase with increasing cone-length was found for these outcomes (Additional file 3: Table S9). Therefore, in a truncated analysis, all cone-lengths up to 10 mm were grouped (*n*=1805) into 10 mm and the risk increase for every mm above 10 mm was analyzed. The aOR for PTD increased by 15% with every mm above 10 mm (Table 6).

**Table 6:** Associations between cone-length and adverse obstetric and neonatal outcomes, truncated analyses of ≤ 10 mm, unadjusted and adjusted multivariable logistic regression analyses

	*n*	OR (95% CI)	*p*	aOR (95% CI) ^a^	*p*
PTD, <37 weeks	187	1.16 (1.09–1.22)	**<0.001**	1.15 (1.09–1.23)	**<0.001**
Spontaneous PTD	149	1.16 (1.09–1.24)	**<0.001**	1.18 (1.10–1.25)	**<0.001**
pPROM	83	1.17 (1.08–1.26)	**<0.001**	1.18 (1.09–1.28)	**<0.000**
PROM, delivery at ≥ 37 weeks	162	1.11 (1.04–1.19)	**0.003**	1.14 (1.05–1.22)	**0.001**
Chorioamnionitis	9	1.06 (0.79–1.42)	0.69	0.99 (0.74–1.34)	0.97
Neonatal sepsis	45	1.16 (1.05–1.28)	**0.003**	1.19 (1.07–1.33)	**0.002**

Statistically significant *p* values in bold type

^a^Analyses adjusted for: year of delivery, maternal age, parity, BMI, marital status, country of birth, infant’s sex, smoking, income, education level and assisted reproduction

*aOR*, adjusted odds ratio; *CI*, confidence interval; *N*, number; *OR*, odds ratio; *pPROM*, preterm prelabor rupture of membranes; *PROM*, prelabor rupture of membranes; *PTD*, preterm delivery

Additional adjustment for the treatment method (LEEP or laser) did not change these findings.

**Fig. 3:**
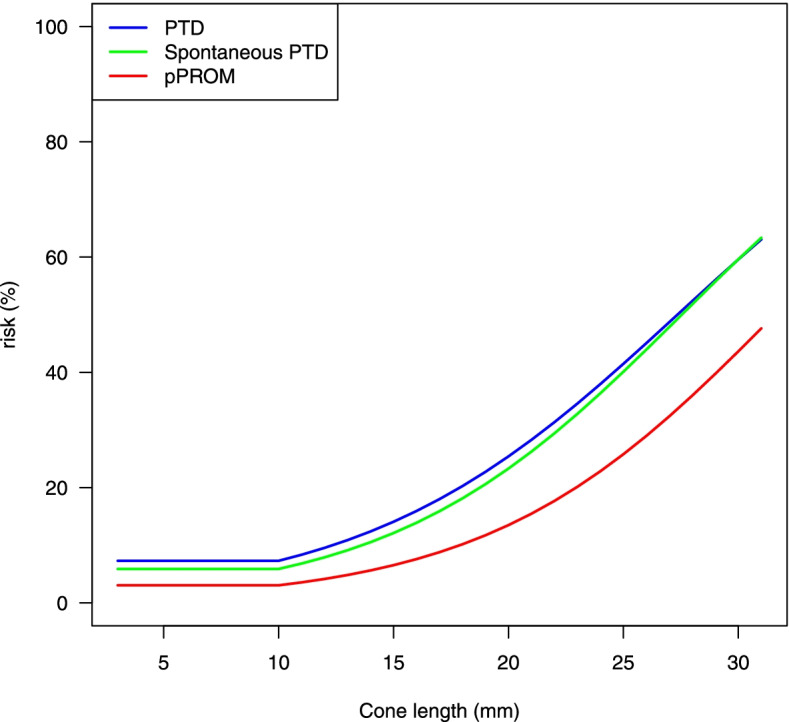
Cone-length and risk of PTD, spontaneous PTD, and pPROM. The risk of PTD, spontaneous PTD, and pPROM increased with cone-length. Risks in relation to cone-lengths (truncated at 10 mm) are estimated using multivariate logistic regression analyses adjusted for age group, parity group, smoking, infant’s sex and time period of delivery and graphed for a group of standardized women (age 23–30, nulliparas, non-smokers, delivery of a male child during the period 2014–2016). PTD, preterm delivery; pPROM, preterm prelabor rupture of the membranes

The risk increases for PTD, spontaneous PTD, and pPROM with cone-length are also illustrated in Fig. 3, showing risk calculation in the truncated model using adjusted multivariable logistic regression.

## Discussion

### Main findings

In this register-based study, based on data from western Sweden in 2008-2016, women who had previously undergone excisional treatment for CIN had an increased risk of PTD, spontaneous PTD, pPROM, and PROM at term, compared to women with a history of normal cytology and women with CIN during pregnancy. This study included a large number of cone-lengths up to 10 mm (*n*=1805). These less extensive treatments were also associated with an increased risk of PTD, spontaneous PTD, and pPROM, an association that remained in comparison to an untreated population with high-grade lesions during pregnancy. The risk of PTD was increased by about 50% in treated women with cone-length ≤ 10 mm, compared to women with normal cervical cytology and women with CIN during pregnancy. The PTD risk was similar for cone-lengths 3-10 mm, thereafter increasing by 15% with each additional millimeter of cone-length.

### Comparison with previous studies and interpretation

Our findings—increased risk of PTD, spontaneous PTD, and pPROM with increasing cone-length, measured before fixation—are consistent with previous studies of cone-length measured after fixation [3, 20, 21, 23]. To the best of our knowledge, this is the first study to compare cone-length at previous treatment to women with untreated CIN during pregnancy. Novel findings of this study are that cone-lengths ≤ 10 mm were associated with increased risk of PTD, spontaneous PTD, and pPROM, including in comparison with women with high-grade CIN, and thus HPV, during pregnancy. Another novel finding was that increasing cone-length was associated with increased risk of PROM at term and increased risk of neonatal sepsis.

In this study population, we could not confirm the increased risks of neonatal mortality, chorioamnionitis, and neonatal sepsis after treatment that were previously found in a larger national Swedish cohort (1999–2016) [8]. However, since our cohort was much smaller this might be due to a lack of power for such uncommon outcomes. Nevertheless, there was an increased risk of chorioamnionitis and neonatal sepsis after treatment in our unadjusted analyses.

This study included treatments from 2008 and onwards, since colposcopists in western Sweden started to record cone-lengths that year, in response to increasing evidence that more extensive treatment might be associated with worse obstetric outcomes [1]. When treating a woman of reproductive age, clinicians try to perform as minor an excision as possible and to avoid unnecessary diathermy after excision. However, treatment must achieve removal of the CIN in order to prevent the development of cancer. Swedish guidelines prescribe a cone-length of 6–9 mm in the most common clinical situation, i.e. when the transformation zone (TZ) is entirely visible on the portio (TZ 1), in order to include the cervical crypts. The mean cone-length in this study, 9.1 mm, reflects adherence to these recommendations. The risk of spontaneous PTD (aOR 2.00, CI 1.70–2.34) after treatment, compared to women with normal cytology, in this cohort, was in the same range as in the previously published national cohort [8] (aOR 2.06, 95% CI 1.95–2.17). The corresponding risk increase of pPROM in this study (aOR 2.63, CI 2.11–3.28) corresponded to aOR 2.36 (95% CI 2.19–2.54), although the national cohort included earlier treatments (60% treated before 2007), undertaken when more radical methods were applied and awareness of obstetric risks was lower. Since detection and treatment of CIN began, excisions have continuously become smaller, preserving cervical tissue. However, this development may possibly have occurred at a cost of diminished reduction in the development of cervical cancer among treated women [28]. This highlights the need for expertise, when it comes to colposcopy, to selection of women for treatment, and to excisional treatment.

The risk estimates for spontaneous PTD after excisional treatment, compared to women with normal cytology, were similar to the results of a cohort study from Denmark (1997–2005) (OR 2.07, 95%CI 1.88–2.27) [5] and to those of a recent Dutch study (2005–2015) (aOR 2.07, 95% CI 1.85–2.33) [13]. However, the risk of spontaneous PTD among previously treated women, compared to women with untreated CIN (aOR 1.95, 95% CI 1.40–2.72), was higher in our study than in the Dutch study (aOR 1.51, 95% CI 1.29–1.76) [13]. Untreated CIN in that study (*n*=5940) included CIN diagnosed in non-pregnant women and was associated with increased risk of spontaneous PTD (aOR 1.38, 95% CI 1.19–1.60), compared to women with normal cytology [13]. In our study, the results pointed in the same direction, i.e., aOR 1.13 (95% CI 0.84–1.51) for spontaneous PTD in women with CIN, compared to women with normal cytology. This was, however, not significant in our smaller cohort.

The Dutch study found an increased risk of spontaneous PTD related to excisions of cervical tissue volumes of 0.50–0.99 cc, measured after fixation, compared to women with CIN. However, information about cone-length was missing and the results are therefore difficult to compare to ours. In contrast to our study, a case-control study from England [20] found no increased risk of PTD when treated women with cone-length <10 mm were compared with untreated women undergoing punch biopsy at colposcopy before or after delivery. Consistent with our result, a Danish study found an increased risk for PTD after cone-length of 10 mm (aOR 1.46 (95% CI 1.11–1.92) compared to untreated women [23]. Other previous studies published on cone-length up to 10 mm have included minimal data [21, 22]. The Danish study of cone-length (LEEP or laser excision, *n*=3605), measured after fixation, found a 6% increase in risk per mm (aOR 1.06, 95% CI 1.03–1.09) [23], lower than in our non-truncated analyses of cone-length (aOR 1.10, 95% CI 1.05–1.15) but within the same CI as in our study. Our truncated analysis, however, showed a higher risk increase, i.e., a 15% increase for each millimeter exceeding 10 mm.

### Strengths and limitations

To the best of our knowledge, this study is the first to investigate cone-length before fixation, measured in a standardized manner. Thus, our results can be related to the clinical situation. Treatments were performed after 2008, with a mean cone-length of 9.1 mm, and can be assumed to represent modern treatment undertaken by colposcopists aware of the increased risk of subsequent PTD. Our study compared treated women both to women with a history of normal cytology as well as to women with untreated CIN during pregnancy. Adjustment for a priori identified confounding factors was performed. For example, assisted reproduction, smoking, and parity had an uneven distribution among the different study groups. However, as this is an observational study, causality cannot be established, and there might still be residual confounding affecting the results. Another limitation is that the small size of the exposure groups, especially the CIN during pregnancy group, limited the power to detect a significantly increased risk for some adverse outcomes.

### Implications and next step for research

The risk of PTD increased with cone-length, but this study shows that small excisions are also associated with an increased risk of spontaneous PTD. The effect of excision size on cervical length measures during pregnancy and, more critical, on the risk of PTD might differ between individual women and possibly depends on the excised volume and/or on the ratio of excised tissue to the total pre-operative cervical volume [16, 24, 42].

Prospective studies are needed in which the cervix is measured before treatment, cone-length and volume are determined before fixation, and women are followed up during pregnancy, including surveillance with standardized ultrasound-measured cervical length [42].

We also suggest more extensive studies comparing women with CIN diagnosed during pregnancy to previously treated women and to women with a history of normal cytology.

Information on excised cone-length, measured at treatment, should be available for risk estimation in subsequent pregnancies, in order to plan surveillance for those at the highest risk of PTD. Risk in the individual after treatment might depend on if other risk factors for PTD also are present. Spontaneous PTD is a multifactorial condition, and only a comprehensive model including major risk factors may present adequate risk estimation for a specific patient [43]. We suggest that future models for individual prediction of spontaneous PTD include cone-length in the excised specimen, together with other risk factors for PTD.

## Conclusions

Excisional treatment is associated with increased risk of PTD, spontaneous PTD, pPROM, and PROM, compared to untreated CIN during pregnancy. The risk is increased but similar related to cone-lengths up to 10 mm, above which it increases with increasing cone-length. Our results indicate that there is no safe cone-length below which there is no increased risk. This highlights the need for well-trained colposcopists to manage women with CIN, including excisional treatment. We recommend that cones be measured in a standardized manner in fresh tissue immediately after treatment. Information about previous treatment for CIN and cone-length should be included in obstetric risk estimation in pregnancies following treatment. Our findings also support the benefit of HPV vaccination programs.

## Supplementary Information

**Additional file 1: Figure S1**. a) and b) Definition of cone-length. c) and d) Standardized measurement of cone-length before fixation in Sweden

**Additional file 2:.** STROBE Checklist. STROBE, Strengthening the Reporting of Observational Studies in Epidemiology

**Additional file 3: Table S1.** ICD-10 diagnosis codes registered in the Swedish Medical Birth Register, leading to exclusion. **Table S2.** Definition of study groups. **Table S3.** Outcome definitions based on ICD-10 diagnosis codes recorded in the Swedish Medical Birth Register. **Table S4.** Classification of cervical cytology and histology. **Table S5a.** Adverse obstetric and neonatal outcomes in cone-length groups, compared to the normal cytology group, unadjusted logistic regression analyses. **Table S5b.** Adverse obstetric and neonatal outcomes in ≤10-mm cone-length groups, compared to the normal cytology group, unadjusted logistic regression analyses. **Table S6.** Adverse obstetric and neonatal outcomes in the ≤10-mm cone-length group, compared to the CIN during pregnancy group, unadjusted and adjusted multivariable analyses. **Table S7.** Adverse obstetric and neonatal outcomes in the ≤10mm cone-length group with high-grade lesions, compared to the CIN during pregnancy subgroup with high-grade lesions, unadjusted and adjusted multivariable analyses. **Table S8.** Associations between cone-length and adverse obstetric and neonatal outcomes, unadjusted and adjusted multivariable logistic regression analyses. **Table S9.** Associations between cone-length for small cones (3-10 mm) and adverse obstetric and neonatal outcomes, unadjusted and adjusted multivariable logistic regression analyses.

## Data Availability

Data cannot be shared publicly. Data from several Swedish national registers have been merged specifically for this project, and we are not allowed to share any individual data. The mandatory Swedish Medical Birth Register and the Swedish Cancer Registry are national datasets and therefore considered to be public property. Access to the data is given only to researchers with permission from a Swedish regional ethical review board (see https://www.etikprovningsmyndigheten.se) and after approval of the research plan by the data managers. Data access requests may be sent to the Swedish National Board of Health and Welfare (https://www.socialstyrelsen.se). After permission from a Swedish regional ethical review board, data researchers can also apply to access data from Statistics Sweden (https://www.scb.se) and the Swedish National Cervical Screening Registry (http://www.nkcx.se/).

## Abbreviations

aOR: Adjusted odds ratio
CIN: Cervical intraepithelial neoplasia
HPV: Human papillomavirus
MBR: The Swedish Medical Birth Register
NKCx: The Swedish National Cervical Screening Registry
pPROM: Preterm prelabor rupture of membranes
PROM: Prelabor rupture of membranes
PTD: Preterm delivery
