# Supplementary material for: Associations between cervical intraepithelial neoplasia during pregnancy, previous excisional treatment, cone-length and preterm delivery: a register-based study from western Sweden
Source: BMC Med. 2022 Feb 22;20:61. doi: 10.1186/s12916-022-02276-6 (PMC8862518; doi:10.1186/s12916-022-02276-6)
Supplement: Supplementary file 1 — Additional file 1: Figure S1. a) and b) Definition of cone-length. c) and d) Standardized measurement of cone-length before fixation in Sweden [file 12916_2022_2276_MOESM1_ESM.pdf]

Figure S1

a) and b) Definition of cone-length
c) and d) Standardized measurement of cone-length before fixation in Sweden:

a)

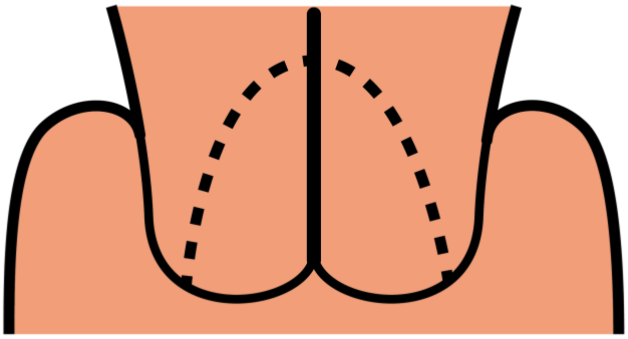

b)

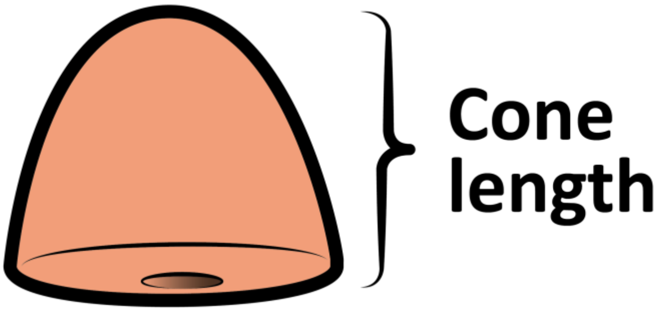

The distance from the external margin to the internal margin of the excised specimen.

c)

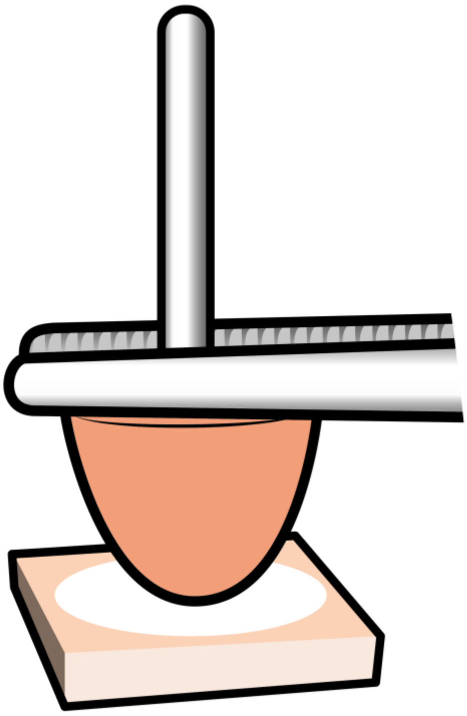

Cone-length is measured by the colposcopist before cutting, montage and fixation in formalin. The endocervical resection area is placed on flat material. A metal pin is placed in the cervical canal. A vertical forceps is placed at the outer cervical os without pressure

d)

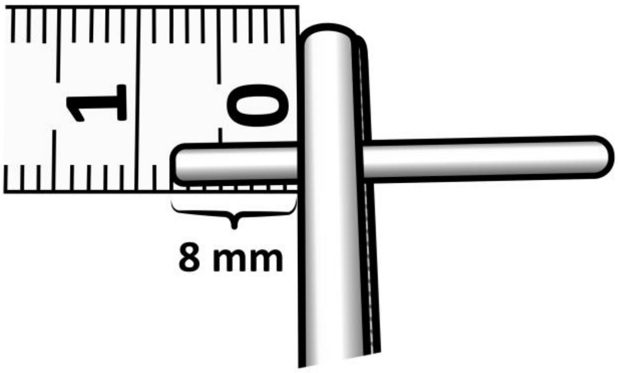

The length of the metal pin enclosed in the specimen is measured.
