# Supplementary material for: Associations between cervical intraepithelial neoplasia during pregnancy, previous excisional treatment, cone-length and preterm delivery: a register-based study from western Sweden
Source: BMC Med. 2022 Feb 22;20:61. doi: 10.1186/s12916-022-02276-6 (PMC8862518; doi:10.1186/s12916-022-02276-6)
Supplement: Supplementary file 2 — Additional file 2:. STROBE Checklist. STROBE, Strengthening the Reporting of Observational Studies in Epidemiology [file 12916_2022_2276_MOESM2_ESM.doc]

**S1 STROBE Checklist**

STROBE Statement—checklist of items that should be included in reports of observational studies

|  | Item No | Recommendation | Section, Paragraph No. | Text from manuscript or authors comment |
| --- | --- | --- | --- | --- |
| **Title and abstract** | 1 | (*a*) Indicate the study’s design with a commonly used term in the title or the abstract | **Title**  **Abstract;** | ***“Associations between cervical intraepithelial neoplasia during pregnancy, previous excisional treatment, cone-length and preterm delivery: a register-based study from western Sweden***  *”*  *“A register-based cohort study in western Sweden”.* |
|  |  | (*b*) Provide in the abstract an informative and balanced summary of what was done and what was found | **Abstract**  -Methods and Results  **Abstract**-Conclusion | ***“Methods***  *A register-based cohort study in western Sweden was conducted linking cervical cytology, histology and treatment data from the Swedish National Cervical Screening Registry to data on obstetric outcomes in singleton pregnancies 2008-2016 from the Swedish Medical Birth Registry. These groups were compared for PTD and other obstetric outcomes: 1) women with one excisional treatment (n=3,250, including a subgroup (n=2,408) with cone-length measured in a standardized manner before fixation, 2) women with untreated CIN diagnosed during pregnancy (n=1,380) and 3) women with a history of normal cytology (n=42,398). Logistic regression analyses were adjusted for socioeconomic and health-related confounders.*  ***Results***  *Treated women had increased risk of PTD (adjusted odds ratio (aOR) 1.60, 95% confidence interval (CI) 1.21-2.12), spontaneous PTD (aOR 1.95, 95% CI 1.40-2.72) and preterm prelabor rupture of membranes (pPROM) (aOR 2.74, 95% CI 1.66-4.51) compared to the CIN during pregnancy group. ORs were similar when compared to the normal cytology group. Risks of these outcomes increased with cone-length. Mean cone-length was 9.1 mm. Cone-length ≤10 mm was associated with increased risk of PTD (aOR 1.41, 95% CI 1.02-1.94), spontaneous PTD (aOR 1.73, 95% CI 1.18-2.54,) and pPROM (aOR 2.44, 95% CI 1.40-4.28), compared to the CIN during pregnancy group. The PTD risk was similar for cone-lengths 3-10 mm, thereafter increasing by 15% with each additional millimeter.”*  *“This study suggests that all excisional treatment, including small cones, are associated with increased risk of PTD and pPROM. Risks increase further with cone-length. In women of reproductive age, clinicians should aim to remove all CIN but minimal healthy cervical tissue. Cone-length should be recorded at treatment, for future prenatal risk estimation. “* |
| Introduction | | |  |  |
| Background/rationale | 2 | Explain the scientific background and rationale for the investigation being reported | **Background**  Para 1 | *“Excisional treatment for cervical intraepithelial neoplasia (CIN) has been associated with preterm delivery (PTD) and preterm prelabor rupture of membranes (pPROM) in subsequent pregnancies”*  *“Studies have yielded contradicting results regarding the impact of excisional treatment on the risk of subsequent PTD. The choice of comparison group seems to affect the effect magnitude. It has been suggested that women with untreated CIN also have increased risk of PTD and that excisional treatment increases the risk further.”*  “*However, the mechanism underlying the increased PTD risk after excisional treatment remains unclear.”*  *“Some previous studies found an increased risk of PTD with increasing cone length at excision and a recent systematic review concluded that risk for PTD increases with cone length.”*  *“It has not been established whether minor excisions (≤10 mm) increase the risk of PTD” To the best of our knowledge, no study has yet examined the effect of different cone lengths, compared to women with untreated CIN during pregnancy.*  *Moreover, associations between cone length and other adverse obstetric outcomes have not been studied in detail.*  *Information about cone length in earlier retrospective studies is based on histopathology reports on formalin-fixed samples, entailing a risk of underestimation due to specimen shrinkage. Ideally, cone length should thus be assessed in fresh tissue. A unique opportunity to study these associations in a population-based manner exists in Sweden. Since 2008, cone length is measured in a standardized way by the colposcopist before fixation, and recorded in the Process Register of the Swedish National Cervical Screening Registry (NKCx/Process). .”* |
| Objectives | 3 | State specific objectives, including any prespecified hypotheses | **Background**  Para 2 | *“This study aimed at exploring the impact of excisional treatment for CIN on the risk of PTD and other adverse obstetric and neonatal outcomes, compared to women with normal cervical cytology history and to women with CIN during pregnancy, as well as at investigating how these risks were related to cone- length at treatment. “* |
| Methods | | |  |  |
| Study design | 4 | Present key elements of study design early in the paper | **Methods** Para1 | “This is a population-based study utilizing data from several Swedish health and quality registers.*”* |
| Setting | 5 | Describe the setting, locations, and relevant dates, including periods of recruitment, exposure, follow-up, and data collection | **Methods**  Para 1  Study population  Para 1  **Figure 1**  **Methods**  Exposures  **Supporting information**  Tables S2  Tables S4 | *“This is a population-based study utilizing data from several Swedish health and quality registers.”*  “All women with singleton births between January 1, 2008 and December 31, 2016 registered in the MBR were identified.”  *“Flowchart of the study population”*  *“A woman with a singleton delivery registered in the MBR 2008-2016 was eligible for inclusion in one of the study groups if the exposure criteria were fulfilled, according to NKCx and Swedish Cancer Register data. Women with treatment recorded in NKCx/Process before 2008 were excluded from all study groups.”*  *“For exact description of study groups, see S2Table.”*  *“Definition of study groups”*  *“Classification of cervical cytology and histology “* |
| Participants | 6 | (*a*) *Cohort study*—Give the eligibility criteria, and the sources and methods of selection of participants.  Describe methods of follow-up  *Case-control study*—Give the eligibility criteria, and the sources and methods of case ascertainment and control selection. Give the rationale for the choice of cases and controls  *Cross-sectional study*—Give the eligibility criteria, and the sources and methods of selection of participants | **Methods**  Study population  Para 1  **Supporting information**  Table S1  **Methods**  Exposures  Para 2  **Supporting Information**  Table S2 | *“All women with singleton births between January 1, 2008 and December 31, 2016 registered in the MBR were identified. Women with a history of chronic inflammatory disease, organ transplantation or human immunodeficiency virus infection were excluded.”*  *“ICD-10 diagnosis codes registered in the Swedish Medical Birth Register, leading to exclusion”.*  *“The study groups were defined as follows:*   1. ***Treated group:*** *cervical excisional treatment performed in western Sweden between Jan 1, 2008 and Dec 31, 2016 and registered in NKCx/Process, and no previous excisional treatment (n= 3,250). The first delivery after treatment was included in the analyses.*   *1b)* ***Cone length group:*** *subgroup of the treated group, treated with loop electrosurgical excision procedure (LEEP) (also known as large loop excision of the transformation zone (LLETZ))) or with laser conization, and with recorded cone length (n= 2,408).*  *Cone-length <3 mm or >50 mm at treatment were excluded due to suspected misreporting.*  *Two women who had undergone cold knife conization (CKC) were excluded to get more homogenous treatment data and facilitate interpretation.*  ***CIN during pregnancy group****: histologically diagnosed CIN or adenocarcinoma in situ (AIS) at cervical biopsy during pregnancy in western Sweden, registered in NKCx/Process, and with no treatment for CIN before the included delivery, (n=1,380). The first eligible delivery was included.*   1. ***Normal cytology group****: lifetime history of exclusively normal cervical cytology results in NKCx/Analysis, and at least one sample taken in western Sweden and registered in NKCx/Process during the three years preceding the included delivery (n=42,398). Only one delivery per woman, chosen at random, was included in the analyses.”*   *“Definition of study groups“* |
|  |  | (*b*) *Cohort study*—For matched studies, give matching criteria and number of exposed and unexposed  *Case-control study*—For matched studies, give matching criteria and the number of controls per case |  |  |
| Variables | 7 | Clearly define all outcomes, exposures, predictors, potential confounders, and effect modifiers. Give diagnostic criteria, if applicable | **Methods**  Exposure  Para 1  **Supporting Information**  Table S2  Table S4  **Methods** Outcomes  Para 1  Para 2  Para 3  **Supporting Information**  Table S3  **Methods**  Background Variables  Para 1  **Results**  Caracterisitics of the study population  Table 1 | Comment; Please see Participants=#6 for study groups  *“Definition of study groups“*  *“Classification of cervical cytology and histology”*  *“The primary outcome was PTD at 22+0 - 36+6 weeks (154-258 days) of gestation, with subanalyses for early PTD (22-33 weeks (154-237 days) of gestation) and very early PTD (22-27 weeks (154-195 days) of gestation). Gestational age was retrieved from the MBR based on the best estimate, i.e., ultrasound determination when available and last menstrual period or estimation of gestational age at the delivery ward in the remaining cases.”*  *“Secondary outcomes were pPROM (determined according to ICD-10 codes in the MBR) and spontaneous PTD (a delivery starting with pPROM or preterm labor, excluding preterm deliveries that started with induction or cesarean section). “*  *“Additional outcomes comprised prelabor rupture of membranes (PROM) in term pregnancies (≥37 weeks of gestation), chorioamnionitis and neonatal sepsis. Furthermore, comparisons were also made between the study groups concerning intrauterine fetal death, neonatal mortality (1-28 days), Apgar score <7 at five minutes, small for gestational age (SGA) (birthweight less than -2 standard deviations (SD) according to Swedish reference curves) and intrapartum fever (see S3 Table for outcome definitions).”*  *“Outcome definitions based on ICD-10 diagnosis codes recorded in the Swedish Medical Birth Register “*  *“Multivariate analyses were adjusted for the following variables retrieved from the prenatal care records in the MBR and SCB registers: year of delivery (2008-2010, 2011-2013, 2014-2016), maternal age at delivery (<23, 23-30, 31-38, >38), body mass index (BMI) (underweight (<18.5), normal-weight (18.5-24.9), overweight (25-29.9), obese (≥30), missing), parity (0,1-3, >3), marital status (cohabiting, single, other, missing), country of birth (Sweden, Europe, Asia, America/Oceania, Africa, other/unknown), infant’s sex (boy/girl), smoking (never, before pregnancy, in early pregnancy only, in the third trimester, missing), highest disposable household income during the three years preceding delivery (population divided into tertiles for every year), education level at delivery (primary, secondary, post-secondary <3 years, post-secondary ≥3 years, missing) and assisted reproduction (yes/no). Furthermore, data on employment at time of delivery, chronic renal disease, diabetes, epilepsy and chronic hypertension were also collected.”*  *“Table 1. Demographics and clinical characteristics in the study groups. “* |
| Data sources/ measurement | 8* | For each variable of interest, give sources of data and details of methods of assessment (measurement). Describe comparability of assessment methods if there is more than one group | **Methods and Material**  Data Sources  Study Population  Exposure  Outcomes  Background Variables  **Tables S2-4** | This is a population-based study utilizing data from several Swedish health and quality registers; (The Swedish Medical Birth Register, The NKCx Analysis and NKCx Process, The Swedish Cancer Register, the Swedish Register of Education, the Total Population Register, the Income and Tax Assessment Register).  Data were linked based on the unique personal identification number held by each resident of Sweden. |
| Bias | 9 | Describe any efforts to address potential sources of bias | **Methods**  Study population  Para 1  **Supporting information**  Table S1  **Methods**  Background Variables  Para 1 | *“singleton births”*  *“Women with a history of chronic inflammatory disease, human immunodeficiency virus infection or organ transplantation were excluded”*  *“ICD-10 diagnosis codes registered in the Swedish Medical Birth Register, leading to exclusion”*  Comment;  Chronic inflammatory disease, HIV infection, earlier organ transplantation and related treatment and multiple pregnancy confers an increased risk for adverse obstetrical outcome. To better study the effect of CIN during pregnancy and earlier treatment for CIN we therefore decided to exclude them.  *“Adjustments were based on a priori knowledge of risk factors for PTD”* |
| Study size | 10 | Explain how the study size was arrived at | **Methods**  Study population  Para 1  Exposure  **Figure 1** | *All women with singleton births between January 1, 2008 and December 31, 2016 registered in the MBR were identified. Women with a history of chronic inflammatory disease, organ transplantation or human immunodeficiency virus infection were excluded (see S1 Table for the International Statistical Classification of Diseases and Related Health Problems -10^th^ revision (ICD-10) codes leading to exclusion).*  *Forty-six thousand eight hundred seventy women had at least one delivery fulfilling the exposure criteria for inclusion in one of the study groups (Fig 1).*  “*A woman with a singleton delivery registered in the MBR 2008-2016 was eligible for inclusion in one of the study groups if the exposure criteria were fulfilled, according to NKCx and Swedish Cancer Register data. Women with treatment recorded in NKCx/Process before 2008 were excluded from all study groups. Furthermore, women with histological diagnoses of CIN2 or of more severe lesions (CIN2+) before 2008 in the NKCx/Analysis and the Swedish Cancer Register were excluded, since they might have undergone excisional treatment (Fig 1).*  *“Flowchart of the study population”* |
| Quantitative variables | 11 | Explain how quantitative variables were handled in the analyses. If applicable, describe which groupings were chosen and why | **Methods**  Outcomes  Para 1  Para 3  Background variables  Para 1 | PTD at 22+0 - 36+6 weeks (154-258 days) of gestation, early PTD (22-33 weeks (154-237 days) of gestation) and very early PTD (22-27 weeks (154-195 days) of gestation).  *“neonatal mortality (1-28 days)”*  *“small for gestational age (SGA) defined as birthweight less than -2 standard deviations (SD) according to the Swedish reference curves “*  Comment; Grouping of potential confounding factors for example; “*BMI (underweight (<18.5), normal weight (18.5-24.9), overweight (25-29.9), obese (≥30), missing)”.* |
| Statistical methods | 12 | (*a*) Describe all statistical methods, including those used to control for confounding | **Methods**  Statistical analyses  Para 2  Para 3  Para 3  Para 4 | *“The normal cytology group, the treated group, and the CIN during pregnancy group were compared, regarding obstetric and neonatal outcomes, by unadjusted and adjusted logistic regression analysis. “*  Comment; To test for threshold effects of cone length at treatment, undadjusted and adjusted logistic regression analyses was performed comparing the reference group to subgroups of cone lengths;  *“Unadjusted and adjusted logistic regression analyses were performed to compare the normal cytology group to the cone length subgroups categorized as follows: 3-5 mm, 6-9 mm, 10 mm,* ***≤*** *10mm, 11-12 mm, 13-15 mm and >15 mm.*  *“Outcomes in the subgroup with cone lengths* ***≤*** *10 mm were also compared to outcomes in the CIN in pregnancy group with unadjusted and adjusted logistic regression analyses*  *“Associations between cone length and PTD, spontaneous PTD, pPROM, PROM, chorioamnionitis and neonatal sepsis were studied with adjusted and unadjusted binary logistic regression in all women with recorded cone lengths, only in women with cone lengths ≤10 mm and in all women after cone lengths < 10 mm had been truncated to 10 mm.*  *Moreover, using the truncated cone lengths, three logistic regression models were fit for the outcomes PTD, spontaneous PTD and pPROM in a standardized woman (age 23-30, nullipara, non-smoker, delivery of a male child during the period 2014-2016), with the left truncated cone length as predictor, and presented graphically.”* |
|  |  | (*b*) Describe any methods used to examine subgroups and interactions | **Methods**  Statistical analyses  Para 2  Para 2  Para 3  Para 3  Para 4 | *“In a subgroup analysis, the treated group with benign histology was compared to the normal cytology group regarding risk of spontaneous PTD and pPROM.”*  *“Furthermore, stratified analyses were used to compare PTD risk, within the CIN during pregnancy group, in women with high-grade lesions to women with low-grade lesions.”*  *“Unadjusted and adjusted logistic regression analyses were performed to compare the normal cytology group to the cone- length subgroups categorized as follows: 3-5 mm, 6-9 mm, 10 mm,* ***≤*** *10mm, 11-12 mm, 13-15 mm and >15 mm.”*  *“Outcomes in the subgroup with cone-lengths* ***≤*** *10 mm were also compared to outcomes in the CIN in pregnancy group with unadjusted and adjusted logistic regression analyses****, including a subgroup analysis exclusively of women with histologically diagnosed high-grade lesions“***  Comment: to find out if there also was a risk increase between 3 and 10 mm a sub-group-analyses of cone-lengths up to 10 mm was done: “ *Associations between cone-length and PTD, spontaneous PTD, pPROM, PROM, chorioamnionitis and neonatal sepsis were studied with adjusted and unadjusted binary logistic regression in all women with recorded cone-lengths,* ***only in women with cone-lengths ≤10*** *mm and in all women after cone-lengths < 10 mm had been truncated to 10 mm.*” |
|  |  | (*c*) Explain how missing data were addressed | **Figure 1**  Table 1  **Methods**  Background variables  Para 1  **Results**  Small for gestational age | *“Flowchart of the study population”*  Comment; women missing gestational age were excluded from the study.  *“Demographics and clinical characteristics in the study groups”.* Comment; Missing background data is shown in Table 1.  Comment; If data for a variable adjusted for in the multivariable regression analyses were missing for any woman a separate category for missing variables were constructed and used,  “*marital status (cohabiting, single, other,* ***missing****),”*  *“*Comment; For the outcome SGA some women had missing data- the number is reported in Tables 2-3. |
|  |  | (*d*) *Cohort study*—If applicable, explain how loss to follow-up was addressed  *Case-control study*—If applicable, explain how matching of cases and controls was addressed  *Cross-sectional study*—If applicable, describe analytical methods *taking account of sampling strategy* | **Results**  Table 1 | Comment; Our definition of study groups resulted in different background factors – see Table 1.  We adjusted for those in the logistic regression analyses. |
|  |  | (*e*) Describe any sensitivity analyses | **Methods**  Statistical Analyses  Para 2  Para 4  **Methods**  Statistical Analyses  Para 2 | Comment; To study the effect of treatment and not eventual effect of disease (CIN) the association of adverse obstetric outcomes was also studied in subgroups - *with benign histology and *with high grade lesions – with unadjusted and adjusted logistic regression.  *“In a subgroup analysis, the treated group with benign histology was compared to the normal cytology group regarding risk for spontaneous PTD and pPROM.“*  *“Outcomes in the subgroup with cone-lengths* ***≤*** *10 mm were also compared to outcomes in the CIN in pregnancy group with unadjusted and adjusted logistic regression analyses, including a subgroup analysis exclusively of women with histologically diagnosed high-grade lesions.”*  Comment; PTD in women with high grade lesions were compared to women with low grade lesions in the CIN during pregnancy group to study if the level of CIN was associated with PTD.  *“Furthermore, stratified analyses were used to compare PTD risk, within the CIN during pregnancy group, in women with high-grade lesions to women with low-grade lesions.”* |

| Results | | |  |  |
| --- | --- | --- | --- | --- |
| Participants | 13* | (a) Report numbers of individuals at each stage of study—eg numbers potentially eligible, examined for eligibility, confirmed eligible, included in the study, completing follow-up, and analysed | **Methods**  Study population  **Results**  Figure 1 | *“Flowchart of the study population“* |
|  |  | (b) Give reasons for non-participation at each stage | **Methods**  **Figure 1**  Study population | *“Flowchart of the study population”* |
|  |  | (c) Consider use of a flow diagram | **Figure 1** | *“Flowchart of the study population”* |
| Descriptive data | 14* | (a) Give characteristics of study participants (eg demographic, clinical, social) and information on exposures and potential confounders | **Results**  Table 1 | *“Demographics and clinical characteristics in the study groups”.* |
|  |  | (b) Indicate number of participants with missing data for each variable of interest | **Results**  Table 1 | *“Demographics and clinical characteristics in the study groups.”* |
|  |  | (c) *Cohort study*—Summarise follow-up time (eg, average and total amount) |  |  |
| Outcome data | 15* | ***Cohort study*—Report numbers of outcome events or summary measures over time** | **Results**  Para3  Table2 | Comment; for the outcomes PTD, spontaneous PTD, pPROM and PROM the results in the total dataset (all singleton deliveries in western Sweden without chronic inflammatory disease- irrespective of cervical test results) is also reported.  *“Table 2. Adverse obstetric and neonatal outcomes in the CIN during pregnancy group and the treated group, compared to the normal cytology group, unadjusted and adjusted multivariable logistic regression analyses “* |
|  |  | ***Case-control study—*Report numbers in each exposure category, or summary measures of exposure** |  |  |
|  |  | ***Cross-sectional study—*Report numbers of outcome events or summary measures** |  |  |
| Main results | 16 | **(*a*) Give unadjusted estimates and, if applicable, confounder-adjusted estimates and their precision (eg, 95% confidence interval). Make clear which confounders were adjusted for and why they were included** | **Methods**  Background variables  **Results**  Tables 2-3  Tables 4a-4b  Tables S5a-5b  **Results**  Para 9  Table 5  **Figure 3**  Table S6    Table S8 | Comment; Description of adjustments  Unadjusted and Adjusted analyses; Description of what was adjusted for is under every table.    Adjusted analyses; Description of what was adjusted for is under every table.  *“Table 4a. Adverse obstetric and neonatal outcomes in cone- length groups, compared to the normal cytology group, adjusted multivariable logistic regression analyses.”*  *“Table 4b. Adverse obstetric and neonatal outcomes in cone- length groups up to 10 mm, compared to the normal cytology group, adjusted multivariable logistic regression analyses”*  Corresponding unadjusted analyses for Tables 4a-4b.  Comment: Unadjusted and adjusted analyses of cone length and obstetric outcomes are in Table 5.  *“in a truncated analysis, all cone-lengths up to 10 mm were grouped (n=1,805) into 10 mm and the risk increase for every mm above 10 mm was analyzed. The aOR for PTD increased by 15% with every mm above 10 mm (Table 5).”*  *“Table 5. Associations between cone-length and adverse obstetric and neonatal outcomes, truncated analyses of ≤ 10mm, unadjusted and adjusted multivariable logistic regression analyses”*  *“Cone-length and risk of PTD, spontaneous PTD and pPROM”*  Comment: Adjusted analyses; Description about what was adjusted for is in Figure legend  *“S6 Table. Adverse obstetric and neonatal outcomes in the ≤10-mm cone-length group, compared to the CIN during pregnancy group, unadjusted and adjusted multivariable analyses”*  Comment: Unadjusted and Adjusted analyses; Description about what was adjusted for is under the table.  *“S8 Table. Associations between cone-length and adverse obstetric and neonatal outcomes, unadjusted and adjusted multivariable logistic regression analyses.”*  Comment: Unadjusted and Adjusted analyses; Description about what was adjusted for is under the table. |
|  |  | (*b*) Report category boundaries when continuous variables were categorized | **Methods**  Outcomes  Para 1 and 3 | Comment; boundaries for PTD, early PTD, very early PTD, neonatal mortality and SGA. |
|  |  | (*c*) If relevant, consider translating estimates of relative risk into absolute risk for a meaningful time period |  |  |
| Other analyses | 17 | Report other analyses done—eg analyses of subgroups and interactions, and sensitivity analyses | **Methods**  **Results**  Para 4  **Results**  Para 5  **Results**  Para 8  Table S7  **Results**  Para 9  Table S9  **Results**  Para 10 | Comment; see under 12e  Comment; Outcomes in women with high grade lesions were compared to women with low grade lesions in the CIN in pregnancy group to study if the level of CIN was associated with adverse outcomes.  *“When women with low-grade lesions were compared to women with high-grade lesions in the CIN during pregnancy group, there was no significant difference in risk of PTD, (aOR 0.90, 95% CI 0.55-1.47, p=0.67).”*  Comment; Results of adjusted subgroup-analyses of treated with benign histology compared to normal cytology group is also described in text:  *“When only women with benign histology in the treated group (n=271) were included, there was still an increased risk of spontaneous PTD (aOR 1.79, 95% CI 1.06 -3.03, p=0.03), as well as of pPROM (aOR 3.04, 95% CI 1.60-5.79, p=0.001), compared to the normal cytology group.”*  *“When treated women with cone-lengths up to 10 mm were compared to the CIN during pregnancy group, we found increased risk of PTD, spontaneous PTD and pPROM (S6 Table);* ***this was also found in the subgroup analyses including only high-grade lesions (S7 Table).”***  *“S7 Table. Adverse obstetric and neonatal outcomes in the ≤10mm cone length group with high-grade lesions, compared to the CIN during pregnancy subgroup with high-grade lesions, unadjusted and adjusted multivariable analyses”*  Comment; The risk for adverse outcomes was similar for cone lengths 3-10 mm.  *“When only cone-lengths 3-10 mm were included in the analyses (n=1,805) no risk increase with increasing cone-length was found for these outcomes”*  *“Associations between cone length for small cones (3-10 mm) and adverse obstetric and neonatal outcomes, unadjusted and adjusted multivariable logistic regression analyses”*    Comment; additional adjustment for treatment method (LEEP or Laser) did not change the result of the cone length analyses.  *“Additional adjustment for treatment method (LEEP or Laser) did not change these findings. “* |
| Discussion | | |  |  |
| Key results | 18 | Summarise key results with reference to study objectives | **Discussion**  Main findings  **Conclusion** | *“In this register-based study, based on data from western Sweden in 2008-2016, women who had previously undergone excisional treatment for CIN had an increased risk of PTD, spontaneous PTD, pPROM and PROM at term, compared to women with a history of normal cytology and women with CIN during pregnancy. This study included a large number of cone lengths up to 10 mm (n=1,805). These less extensive treatments were also associated with an increased risk of PTD, spontaneous PTD and pPROM, an association that remained in comparison to an untreated population with high-grade lesions during pregnancy. The risk of PTD was increased by about 50% in treated women with cone length ≤ 10 mm, compared to women with normal cervical cytology and women with CIN during pregnancy. The PTD risk was similar for cone lengths 3-10 mm, thereafter increasing by 15% with each additional millimeter of cone length.”*  *“Excisional treatment is associated with increased risk of PTD, spontaneous PTD, pPROM and PROM, compared to untreated CIN during pregnancy. The risk is increased but similar related to cone lengths up to 10 mm, above which it increases with increasing cone-length. Our results indicate that there is no safe cone length below which there is no increased risk.”* |
| Limitations | 19 | Discuss limitations of the study, taking into account sources of potential bias or imprecision. Discuss both direction and magnitude of any potential bias | **Discussion**  Strengths and Limitations | *“as this is an observational study, causality cannot be established and there might still be residual confounding affecting the results. Another limitation is that the small size of the exposure groups, especially the CIN during pregnancy group, limited the power to detect a significantly increased risk for some adverse outcomes.”* |
| Interpretation | 20 | Give a cautious overall interpretation of results considering objectives, limitations, multiplicity of analyses, results from similar studies, and other relevant evidence | **Discussion** Comparision with previous studies and interpretation  **Limitations**  **Conclusion** | *“Our findings ─ increased risk of PTD, spontaneous PTD and pPROM with increasing cone length, measured before fixation ─ are consistent with previous studies of cone length measured after fixation”*  *“Novel findings of this study are that cone lengths ≤ 10 mm were associated with increased risk of PTD, spontaneous PTD and pPROM, including in comparison with women with high-grade CIN, and thus HPV, during pregnancy. Another novel finding was that increasing cone length was associated with increased risk of PROM at term and increased risk of neonatal sepsis.”*  Comment: Compared to a national Swedish study:  *“In this study population, we could not confirm the increased risks of neonatal mortality, chorioamnionitis and neonatal sepsis after treatment that were previously found in a larger national Swedish cohort (1999-2016)”*  *“The risk of spontaneous PTD (aOR 2.00, CI 1.70-2.34) after treatment, compared to women with normal cytology, in this cohort was in the same range as in the previously published national cohort”* “*The corresponding risk increase of pPROM in this study (aOR 2.63, CI 2.11-3.28) corresponded to aOR 2.36 (95% CI 2.19-2.54), although the national cohort included earlier treatments (60% treated before 2007), undertaken when more radical methods were applied and awareness of obstetric risks was lower*.”  Comment; compared to two Danish studies, a Dutch study and a study from England;  *“The risk estimates for spontaneous PTD after excisional treatment, compared to women with normal cytology, were similar to the results of a cohort study from Denmark (1997-2005) (OR 2.07, 95%CI 1.88-2.27 and to those of a recent Dutch study (2005-2015) (aOR 2.07, 95% CI 1.85-2.33”* “*However, the risk of spontaneous PTD among previously treated women, compared to women with untreated CIN (aOR 1.95, 95% CI 1.40-2.72), was higher in our study than in the Dutch study (aOR 1.51, 95% CI 1.29-1.76)”* *“Untreated CIN in that study (n=5,940) included CIN diagnosed in non-pregnant women and was associated with increased risk of spontaneous PTD (aOR 1.38, 95% CI 1.19-1.60), compared to women with normal cytology. In our study, the results pointed in the same direction,”*  *“In contrast to our study, a case-control study from England found no increased risk of PTD when treated women with cone length <10 mm were compared with untreated women undergoing punch biopsy at colposcopy before or after delivery.”*  “*Consistent with our result a Danish study found an increased risk for PTD after cone length of 10 mm (aOR 1.46 (95% CI 1.11-1.92) compared to untreated women”*  *“Other previous studies published on cone length up to 10 mm have included minimal data”*  “*The Danish study of cone length (LEEP or laser excision, n=3,605), measured after fixation, found a 6% increase in risk per mm (aOR 1.06, 95% CI 1.03-1.09). lower than in our non-truncated analyses of cone length (aOR 1.10, 95% CI 1.05-1.15) but within the same CI as in our study. Our truncated analysis, however, showed a higher risk increase, i.e. a 15% increase for each millimeter exceeding 10mm. “*  *“Another limitation is that the small size of the exposure groups, especially the CIN during pregnancy group, limited the power to detect a significantly increased risk for some adverse outcomes”.*  *“Excisional treatment is associated with increased risk of PTD, spontaneous PTD, pPROM and PROM, compared to untreated CIN during pregnancy. The risk is increased but similar related to cone-lengths up to 10 mm, above which it increases with increasing cone-length. Our results indicate that there is no safe cone*-*length below which there is no increased risk. This highlights the need for well-trained colposcopists to manage women with CIN, including excisional treatment. We recommend that cones be measured in a standardized manner in fresh tissue immediately after treatment. Information about previous treatment for CIN and cone length should be included in obstetric risk estimation in pregnancies following treatment. Our findings also support the benefit of HPV vaccination programs.”* |
| Generalisability | 21 | Discuss the generalisability (external validity) of the study results | **Discussion**  Strengths and limitations | Comment; This study is a population-based study. The surgical treatment methods and the definition of exposure in this study are used world-wide and so are also the definitions of outcomes in this study (based on ICD-10). Treatment and delivery were between 2008-2016 and the study includes thorough adjustment for background factors. This support generalizability.  *“To the best of our knowledge, this study is the first to investigate cone length before fixation, measured in a standardized manner. Thus, our results can be related to the clinical situation. Treatments were performed after 2008, with a mean cone length of 9.1 mm, and can be assumed to represent modern treatment undertaken by colposcopists aware of the increased risk of subsequent PTD. Our study compared treated women both to women with a history of normal cytology as well as to women with untreated CIN during pregnancy. Thorough adjustment for potentially confounding background factors was performed.”* |
| Other information | | |  |  |
| Funding | 22 | Give the source of funding and the role of the funders for the present study and, if applicable, for the original study on which the present article is based |  | Funding: JW has received research grants from Østfold Hospital Trust, Norway (nr 16/04196), and Hjalmar Svenssons forskningsfond, Sweden (nr HJSV2020079 and nr HJSV2021036). VS has received research grants from Wilhelm och Martina Lundgren Vetenskapsfond (2016-1005) and Fru Mary von Sydows, född Wijk, donationsfond (nr 1016). “The funders had no role in study design, data collection and analysis, decision to publish, or preparation of the manuscript.” |

*Give information separately for cases and controls in case-control studies and, if applicable, for exposed and unexposed groups in cohort and cross-sectional studies.

**Note:** An Explanation and Elaboration article discusses each checklist item and gives methodological background and published examples of transparent reporting. The STROBE checklist is best used in conjunction with this article (freely available on the Web sites of PLoS Medicine at http://www.plosmedicine.org/, Annals of Internal Medicine at http://www.annals.org/, and Epidemiology at http://www.epidem.com/). Information on the STROBE Initiative is available at [www.strobe-statement.org](http://www.strobe-statement.org/).
