# Supplementary material for: Associations between cervical intraepithelial neoplasia during pregnancy, previous excisional treatment, cone-length and preterm delivery: a register-based study from western Sweden
Source: BMC Med. 2022 Feb 22;20:61. doi: 10.1186/s12916-022-02276-6 (PMC8862518; doi:10.1186/s12916-022-02276-6)
Supplement: Supplementary file 3 — Additional file 3: Table S1. ICD-10 diagnosis codes registered in the Swedish Medical Birth Register, leading to exclusion. Table S2. Definition of study groups. Table S3. Outcome definitions based on ICD-10 diagnosis codes recorded in the Swedish Medical Birth Register. Table S4. Classification of cervical cytology and histology. Table S5a. Adverse obstetric and neonatal outcomes in cone-length groups, compared to the normal cytology group, unadjusted logistic regression analyses. Table S5b. Adverse obstetric and neonatal outcomes in ≤10-mm cone-length groups, compared to the normal cytology group, unadjusted logistic regression analyses. Table S6. Adverse obstetric and neonatal outcomes in the ≤10-mm cone-length group, compared to the CIN during pregnancy group, unadjusted and adjusted multivariable analyses. Table S7. Adverse obstetric and neonatal outcomes in the ≤10mm cone-length group with high-grade lesions, compared to the CIN during pregnancy subgroup with high-grade lesions, unadjusted and adjusted multivariable analyses. Table S8. Associations between cone-length and adverse obstetric and neonatal outcomes, unadjusted and adjusted multivariable logistic regression analyses. Table S9. Associations between cone-length for small cones (3-10 mm) and adverse obstetric and neonatal outcomes, unadjusted and adjusted multivariable logistic regression analyses. [file 12916_2022_2276_MOESM3_ESM.doc]

**Table S1. ICD-10 diagnosis codes registered in the Swedish Medical Birth Register, leading to exclusion**

| **Inflammatory arthritis and systemic inflammatory disease** |
| --- |
| M05, M05.0, M05.1, M05.2, M05.3, M05.8, M05.8A, M05.8B, M05.8C. M058D, M058F, M058G, M058H, M05.8L, M05.8M, M05.8N, M05.8X, M05.9, M05.9L, M05.9M, M05.9N  M06.0, M06.0L, M060M, M06.0N, M06.1, M06.2, M06.3, M06.4, M06.8, M06.8L, M06.8L, M06.8M, M06.9, M06.9A, M06.9B, M06.9C, M06.9D, M06.9F, M06.9G, M06.9H, M06.9X  M08.0, M08.0B, M08.0C, M08.0D, M08.0F, M08.0G, M08.0H, M08.0X, M08.1, M08.2, M08.2A, M08.2B, M08.3, M08.4, M08.4A, M08.4B, M08.8, M08.9  M30.0, M30.1, M30.2, M30.3, M30.8  M31.0, M31.1, M31.3, M31.4, M31.5, M31.6, M31.7, M31.8  M32, M32.0, M32.1, M32.8, M32.8A, M32.8B, M32.8C, M32.8D, M32.8W, M32.9  M33.0, M33.1, M33.2, M33.9  M34.0, M34.1, M34.2, M34.8, M34.9  M35.0, M35.0A, M35.0B, M35.1, M35.2, M35.3  K75.4 |
| **Inflammatory bowel disease** |
| K50.0, K50.1, K50.8, K50.9  K51, K51.0, K51.2, K51.3, K51.4, K51.5, K51.8, K51.9 |
| **Organ transplantation** |
| Z94.0, Z94.1, Z94.2, Z94.3, Z94.4, Z94.6, Z94.8, Z94.9 |
| **HIV infection** |
| O98.7  B20, B20.1, B20.4  B22  B23.1, B23.2, B23.8  B24 |

Abbreviations: ICD=International Statistical Classification of Diseases and Related Health Problems; HIV= human immunodeficiency virus

**Table S2. Definition of study groups**

| **Study group** | **Exposures as inclusion criteria^1^** | **Additional inclusion criteria^1^** | **Comment** |
| --- | --- | --- | --- |
| **Treated group** | Excisional treatment 2008-2016 in western Sweden, registered in NKCx/Process before delivery  AND  no previous treatment | No excisional treatment between 2002-2008 recorded in NKCx/Process  No CIN2+ (CIN2, CIN3, AIS, cancer) in cervical  histology recorded in NKCx/Analysis or the National Swedish Cancer Register prior to 2008 | No previous treatment is presumed since there is:  -no previous excisional treatment recorded in NKCx/Process before the included treatment.  -no histologically diagnosed CIN2+ in NKCx/Analysis or the National Swedish Cancer Register before 2008. |
| **Cone-length group** | Excisional treatment with. LEEP or laser excision 2008-2016 in western Sweden, registered in NKCx/Process before delivery  AND  no previous treatment  AND  recorded cone-length >2 mm and <50 mm | No excisional treatment between 2002-2008 recorded in NKCx/Process  No CIN2+ (CIN2, CIN3, AIS, cancer) in cervical  histology recorded in NKCx/Analysis or the National Swedish Cancer Register prior to 2008 | No previous treatment is presumed since there is:  -no previous excisional treatment recorded in NKCx/Process before the included treatment.  -no histologically diagnosed CIN2+ in NKCx/Analysis or the National Swedish Cancer Register before 2008. |
| **CIN during pregnancy group** | Low-grade lesions (CIN1) or high-grade lesions (CIN2, CIN3, AIS) in cervical biopsy, histologically diagnosed during pregnancy in western Sweden, registered in NKCx/Process | No excisional treatment between 2002-2008 recorded in NKCx/Process  No CIN2+ (CIN2, CIN3, AIS, cancer) in cervical  histology recorded in NKCx/Analysis or the National Swedish Cancer Register prior to 2008 | No previous treatment is presumed since there is:  -no previous excisional treatment recorded in NKCx/Process before the included treatment.  -no histologically diagnosed CIN2+ in NKCx/Analysis or the National Swedish Cancer Register before 2008. |
| **Normal cytology group** | Exclusively normal cervical cytology results registered in the NKCx/Analysis  AND  Samples taken least every fifth year from age 23 years^2^ until end of study period or age 45 years  AND  At least one sample taken in western Sweden 3 years preceding the included delivery | No positive cervical HPV test between 6 months before conception and delivery | Women who had participated in the Swedish cervical screening program and had no recorded abnormal cervical cytology sample in Sweden. |

^1^ Based on cytology, histology and/or registered treatment in the Swedish National Cervical Screening Registry and/or the Swedish Cancer Register.

^2^ Women in Sweden are invited for cervical screening every third year, beginning from 23 years of age.

Abbreviations: AIS=adenocarcinoma in situ; CIN=cervical intraepithelial neoplasia; HPV=human papillomavirus; NKCx, The Swedish National Cervical Screening Registry

**Table S3. Outcome definitions based on ICD-10 diagnosis codes recorded in the Swedish Medical Birth Register**

| ICD-10 codes | Additional criteria |
| --- | --- |
| **pPROM** | |
| O42.  O42.0, O42.1, O42.2, O42.9  P01.1 | Gestational age ≤ 262 days^1^ otherwise classified as PROM |
| **PROM** | |
| O756.6, O75.6A, O75.6B, O75.6X | Gestational age >258 days otherwise classified as pPROM |
| **Chorioamnionitis** | |
| O41.1  P02.7  O75.3 |  |
| **Intrapartum fever** | |
| O75.2 |  |
| **Neonatal sepsis** |  |
| P36.0-P36.9  A40, A400, A401, A403, A408, A409  A41, A410, A411, A412, A413, A414, A415, A418, A419 |  |

^1^Gestational age ≤ 262 days in combination with diagnosis of pPROM was considered to be correctly diagnosed

Abbreviations: ICD=International Statistical Classification of Diseases and Related Health Problems; pPROM=preterm prelabor rupture of membranes; PROM=prelabor rupture of membranes

**Table S4. Classification of cervical cytology and histology**

| SNOMED  from NKCx/Analysis  and  NKCx/Process | Classification used in this study, based on classification in NKCx/Process | Original classification of histology in NKCx/Process | SNOMED  from  NKCx/Analysis | | Swedish Cancer Register |
| --- | --- | --- | --- | --- | --- |
| Used for  -inclusion in normal cytology group | Used for  - description of histology in cone in treated group  - inclusion in CIN during pregnancy group |  | Used for  -exclusion from all groups (due to possible previous treatment) | | Used for  -exclusion from all groups (due to possible previous treatment) |
| ***Cytology*** | ***Histology*** | ***Histology*** | ***Histology*** | | ***Histology**** |
| M00110 = Normal | Benign | Benign |  |  |  |
|  | Dysplasia not further specified or signs of HPV infection | Premalignant, suspected |  |  |  |
|  |  | Premalignant UNS |  |  |  |
|  |  | Signs of HPV infection |  |  |  |
|  | Low-grade | Low-grade |  |  |  |
|  | High-grade | High-grade | HSIL/moderate atypia  CIN 2 | M74007 |  |
|  |  |  | HSIL/severe atypia  CIN3/CIS | M80702 | 144/b |
|  |  |  | AIS | M81402 | 094 |
|  |  | Suspected cancer |  |  |  |
|  | Cancer | Cancer | Squamous cell carcinoma | M80703 | 146 |
|  |  |  | Adenocarcinoma | M81403 | 096 |

^*^ Also including ICD7=171

AIS, adenocarcinoma in situ, CIN=cervical intraepithelial neoplasia; CIS, cancer in situ; HSIL, high-grade squamous intraepithelial lesion; LSIL, low-grade squamous intraepithelial lesion; NKCx, The Swedish National Cervical Screening Registry; SNOMED, Systematized Nomenclature of Medicine; UNS, unspecified

**Table S5a. Adverse obstetric and neonatal outcomes in cone-length groups, compared to the normal cytology group, unadjusted logistic regression analyses**

|  | Normal cytology group  N=42,398 | Treated, cone-length ≤ 10mm  N=1,805 | | | Treated, cone-length  11-12mm  N=361 | | | Treated, cone-length  13-15  N=174 | | | Treated, cone-length  >15  N=68 | | |
| --- | --- | --- | --- | --- | --- | --- | --- | --- | --- | --- | --- | --- | --- |
| Outcomes | N  (%) | N (%) | OR (95% CI) | p | N (%) | OR (95% CI) | p | N  (%) | OR (95% CI) | p | N  (%) | OR (95% CI) | p |
| PTD, <37 weeks | 1,794  (4.2) | 119 (6.6) | 1.60 (1.32-1.94) | **<0.001** | 33 (9.1) | 2.28 (1.59-3.27) | **<0.001** | 20 (11.5) | 2.94 (1.84-4.70) | **<0.001** | 15 (22.1) | 6.41 (3.60-11.39) | **<0.001** |
| Spontaneous PTD | 1,264  (3.0) | 93 (5.2) | 1.77 (1.42-2.19) | **<0.001** | 27 (7.5) | 2.63 (1.77-3.91) | **<0.001** | 17 (9.8) | 3.52 (2.13-5.83) | **<0.001** | 12 (17.6) | 6.97 (3.73-13.04) | **<0.001** |
| pPROM | 479  (1.1) | 53 (2.9) | 2.65 (1.99-3.53) | **<0.001** | 15 (4.2) | 3.79 (2.25-6.41) | **<0.001** | 6 (3.4) | 3.13 (1.38-7.09) | **0.006** | 9 (13.2) | 13.35 (6.58-27.08) | **<0.001** |
| PROM, delivery at ≥ 37 weeks | 2,084  (5.1) | 113 (6.7) | 1.33 (1.09-1.62) | **0.005** | 30 (9.1) | 1.86 (1.28-2.72) | **0.001** | 9  (5.8) | 1.15 (0.58-2.25) | 0.69 | 10 (18.9) | 4.30 (2.16-8.57) | **<0.001** |
| Chorioamnionitis | 97  (0.2) | 7 (0.4) | 1.70 (0.79-3.66) | 0.18 | 1 (0.3) | 1.21 (1.17-8.71) | 0.85 | 0 | - | - | 1 (1.5) | 6.51 (0.90-47.36) | 0.064 |
| Neonatal sepsis | 591  (1.4) | 31 (1.7) | 1.24 (0.86-1.78) | 0.25 | 5 (1.4) | 0.99 (0.41 -2.41) | 0.99 | 3 (1.7) | 1.24 (0.40-3.90) | 0.71 | 6 (8.8) | 6.85 (2.95-15.89) | **<0.001** |

Statistically significant *p*-values in bold type

CI, confidence interval; CIN, cervical intraepithelial neoplasia; N, number; OR, odds ratio; pPROM, preterm prelabor rupture of membranes; PROM, prelabor rupture of membranes; PTD, preterm delivery.

**Table S5b. Adverse obstetric and neonatal outcomes in ≤10-mm cone-length groups, compared to the normal cytology group, unadjusted logistic regression analyses**

|  | Normal cytology  group  N=42,398 | Treated, cone-length < 6mm  N=212 | | | Treated, cone-length  6-9 mm  N=1219 | | | Treated, cone-length  10 mm  N=374 | | |
| --- | --- | --- | --- | --- | --- | --- | --- | --- | --- | --- |
| Outcomes | N  (%) | N (%) | OR(95% CI) | p | N (%) | OR(95% CI) | p | N (%) | OR(95% CI) | p |
| PTD <37 weeks | 1,794 (4.2) | 16 (7.5) | 1.85 (1.11-3.08) | **0.019** | 77 (6.3) | 1.53 (1.21-1.93) | **<0.001** | 26 (7.0) | 1.69 (1.13-2.53) | **0.010** |
| Spontaneous PTD | 1,264 (3.0) | 12 (5.7) | 1.95 (1.09-3.51) | **0.025** | 61 (5.0) | 1.71 (1.32-2.23) | **<0.001** | 20 (5.3) | 1.84 (1.17-2.90) | **0.009** |
| pPROM | 479  (1.1) | 7 (3.3) | 2.99 (1.40-6.38) | **0.005** | 36 (3.0) | 2.66 (1.89-3.76) | **<0.001** | 10 (2.7) | 2.40 (1.27-4.54) | **0.007** |
| PROM in deliveries at ≥ 37 weeks | 2,084 (5.1) | 11 (5.6) | 1.10 (0.60-2.02) | 0.76 | 79 (6.9) | 1.37 (1.09-1.73) | **0.008** | 23 (6.6) | 1.31 (0.86-2.00) | 0.22 |
| Chorioamnionitis | 97  (0.2) | 0 | - | - | 4 (0.3) | 1.44 (0.53-3.91) | 0.48 | 3 (0.8) | 3.53 (1.11-11.18) | **0.032** |
| Neonatal sepsis | 591  (1.4) | 3 (1.4) | 1.02 (0.32-3.18) | 0.98 | 22 (1.8) | 1.30 (0.85-2.00) | 0.23 | 6 (1.6) | 1.15 (0.51-2.60) | 0.73 |

Statistically significant *p*-values in bold type.

CI, confidence interval; CIN, cervical intraepithelial neoplasia; N, number; OR, odds ratio; pPROM, preterm prelabor rupture of membranes; PROM, prelabor rupture of membranes; PTD, preterm delivery.

**Table S6. Adverse obstetric and neonatal outcomes in the ≤10-mm cone-length group, compared to the CIN during pregnancy group, unadjusted and adjusted multivariable analyses**

|  | CIN during pregnancy group  N=1,380 | Treated,  cone-length  **≤**10mm  N=1,711 | Unadjusted | | Adjusted^1^ | |
| --- | --- | --- | --- | --- | --- | --- |
| Outcome | n (%) | n (%) | OR (95% CI) | p-value | aOR (95% CI) | p-value |
| PTD, <37 weeks | 74 (5.4) | 115 (6.7) | 1.27 (0.94-1.72) | 0.12 | 1.41 (1.02-1.94) | **0.038** |
| Spontaneous PTD | 49 (3.6) | 89 (5.2) | 1.49 (1.04-2.13) | **0.028** | 1.73 (1.18-2.54) | **0.005** |
| pPROM | 20 (1.4) | 52 (3.0) | 2.13 (1.27-3.59) | **0.004** | 2.44 (1.40-4.28) | **0.002** |
| PROM, delivery at ≥ 37 weeks | 69 (5.3) | 111 (7.0) | 1.34 (0.98-1.83) | 0.06 | 1.35 (0.97-1.88) | 0.08 |
| Chorioamnionitis | 5 (0.4) | 7 (0.4) | 1.13 (0.36-3.57) | 0.84 | 0.89 (0.25-3.17) | 0.86 |
| Neonatal sepsis | 22 (1.6) | 29 (1.7) | 1.06 (0.61-1.86) | 0.83 | 1.09 (0.60-1.99) | 0.77 |

Statistically significant *p*-values in bold type.

^1^ Analyses adjusted for: year of delivery, maternal age, parity, BMI, marital status, country of birth, infant’s sex, smoking, income, education level and assisted reproduction.

aOR, adjusted odds ratio; CI, confidence interval; CIN, cervical intraepithelial neoplasia; mm, millimeter; N, number; OR, odds ratio; pPROM, preterm prelabor rupture of membranes; PROM, prelabor rupture of membranes; PTD, preterm delivery;

**Table S7. Adverse obstetric and neonatal outcomes in the ≤10mm cone-length group with high-grade lesions, compared to the CIN during pregnancy subgroup with high-grade lesions, unadjusted and adjusted multivariable analyses**

|  | CIN during pregnancy group  with high-grade lesions  N=830 | Treated, cone-length  **≤** 10 mm,  with high-grade lesions  N=1,261 | Unadjusted | | Adjusted^1^ | |
| --- | --- | --- | --- | --- | --- | --- |
|  | n (%) | n (%) | OR (95% CI) | p-value | aOR (95% CI) | p-value |
| PTD, <37 weeks | 42 (5.1) | 88 (7.0) | 1.41 (0.97-2.06) | 0.077 | 1.52 (1.01-2.27) | **0.044** |
| Spontaneous PTD | 28 (3.4) | 69 (5.5) | 1.66 (1.06-2.60) | **0.027** | 1.86 (1.15-3.01) | **0.011** |
| pPROM | 12 (1.4) | 37 (2.9) | 2.06 (1.07-3.98) | **0.031** | 2.32 (1.14-4.73) | **0.021** |
| PROM, delivery at ≥ 37 weeks | 47 (6.0) | 80 (6.8) | 1.15 (0.80-1.67) | 0.45 | 1.09 (0.73-1.62) | 0.68 |
| Chorioamnionitis | 4 (0.5) | 6 (0.5) | 0.99 (0.28-3.51) | 0.98 | 0.61 (0.14-2.68) | 0.52 |
| Neonatal sepsis | 13 (1.6) | 23 (1.8) | 1.17 (0.59-2.32) | 0.66 | 1.18 (0.55-2.52) | 0.67 |

Statistically significant *p*-values in bold type.

^1^ Analyses adjusted for: year of delivery, maternal age, parity, BMI, marital status, country of birth, infant’s sex, smoking, income, education level and assisted reproduction.

aOR, adjusted odds ratio; CI, confidence interval; CIN, cervical intraepithelial neoplasia; mm, millimeter; N, number; OR, odds ratio; pPROM, preterm prelabor rupture of membranes; PROM, prelabor rupture of membranes; PTD, preterm delivery

**Table S8. Associations between cone-length and adverse obstetric and neonatal outcomes, unadjusted and adjusted multivariable logistic regression analyses**

| Outcome | N=2,408 | OR (95% CI) | p | aOR (95% CI) ^1^ | p |
| --- | --- | --- | --- | --- | --- |
| PTD, <37 weeks | 187 | 1.11 (1.06-1.15) | **<0.001** | 1.10 (1.05-1.15) | **<0.001** |
| Spontaneous PTD | 149 | 1.12 (1.07-1.17) | **<0.001** | 1.12 (1.07-1.18) | **<0.001** |
| pPROM | 83 | 1.11 (1.05-1.18) | **0.001** | 1.12 (1.05-1.19) | **0.001** |
| PROM, delivery at ≥ 37 weeks | 162 | 1.07 (1.02-1.12) | **0.009** | 1.08 (1.03-1.14) | **0.003** |
| Chorioamnionitis | 9 | 1.11 (0.93-1.32) | 0.25 | 1.08 (0.89-1.31) | 0.43 |
| Neonatal sepsis | 45 | 1.11 (1.02-1.20) | **0.014** | 1.12 (1.03-1.23) | **0.009** |

Statistically significant *p*-values in bold type.

^1^ Analyses adjusted for: year of delivery, maternal age, parity, BMI, marital status, country of birth, infant’s sex, smoking, income, education level and assisted reproduction.

aOR, adjusted odds ratio; CI, confidence interval; N, number; OR, odds ratio; pPROM, preterm prelabor rupture of membranes; PROM, prelabor rupture of membranes; PTD, preterm delivery.

**Table S9. Associations between cone-length for small cones (3-10 mm) and adverse obstetric and neonatal outcomes, unadjusted and adjusted multivariable logistic regression analyses**

| Outcome | N=1,805 | OR (95% CI) | p | aOR (95% CI) | p |
| --- | --- | --- | --- | --- | --- |
| PTD, <37 weeks | 119 | 1.03 (0.92-1.15) | 0.65 | 1.01 (0.90-1.13) | 0.88 |
| Spontaneous PTD | 93 | 1.06 (0.93-1.20) | 0.40 | 1.04 (0.92-1.18) | 0.53 |
| pPROM | 53 | 1.00 (0.85-1.18) | 0.96 | 1.00 (0.85-1.18) | 0.98 |
| PROM, delivery at ≥ 37 weeks | 113 | 1.02 (0.91-1.14) | 0.76 | 1.03 (0.92-1.16) | 0.61 |
| Chorioamnionitis | 7 | 1.75 (0.96-3.20) | 0.07 | 2.36 (0.97-5.76) | 0.06 |
| Neonatal sepsis | 31 | 1.06 (0.85-1.31) | 0.61 | 1.07 (0.85-1.33) | 0.57 |

^1^ Analyses adjusted for: year of delivery, maternal age, parity, BMI, marital status, country of birth, infant’s sex, smoking, income, education level and assisted reproduction.

aOR, adjusted odds ratio; CI, confidence interval; N, number; OR, odds ratio; pPROM, preterm prelabor rupture of membranes; PROM, prelabor rupture of membranes; PTD, preterm delivery.
